# Screening, Diversity, and Characterization of Fungal Endophytes Isolated From the Halophyte *Limonium axillare* and the Potential of Biocontrol Antagonists Against *Fusarium oxysporum*

**DOI:** 10.1002/pld3.70026

**Published:** 2025-03-24

**Authors:** Fedae Alhaddad, Mohammed Abu‐Dieyeh, Samir Jaoua, Mohammad A. Al‐Ghouti, Roda Al‐Thani, Talaat Ahmed

**Affiliations:** ^1^ Biological Science Program, Department of Biological and Environmental Sciences, College of Arts and Sciences Qatar University Doha Qatar; ^2^ Environmental Science Program, Department of Biological and Environmental Sciences, College of Arts and Sciences Qatar University Doha Qatar; ^3^ Environmental Science Center, Research and Graduate Studies Qatar University Doha Qatar

**Keywords:** biocontrol, fungal endophytes, halophyte, promoting growth

## Abstract

Halophytes, plants that thrive in high‐salinity environments, host unique microbial communities, including fungal endophytes, which contribute to plant growth and pathogen resistance. This study aimed to isolate, identify, and evaluate the antagonistic potential of fungal endophytes from the halophytic plant *Limonium axillare*, collected from both inland and coastal habitats. Fungal endophytes were isolated, identified via molecular techniques, and tested for antagonistic activity against phytopathogenic fungi using dual‐culture assays. The results showed a diverse range of fungal endophytes, with *Aspergillus* and *Cladosporium* being the dominant genera. A total of 152 endophytic fungi were isolated from both locations, with 95 isolates coming from coastal plants and 57 from inland species. The isolates exhibited varying degrees of antagonistic activity against phytopathogens, highlighting their potential role in plant protection. Further research is needed to clarify these interactions' mechanisms and investigate their practical applications in agriculture. An endophytic isolate of *Aspergillus terreus* strain ((AL10) lim10qu) (ON210104.1) exhibited potent in vitro antifungal activity against *Fusarium oxysporum*, a pathogenic fungus affecting tomato plants. Greenhouse experiments demonstrated that the fungus significantly increased both the length of tomato seedlings and the overall plant biomass. Both laboratory‐based (in vitro) and field‐based (in vivo) evaluations of the strain ((AL10) lim10qu) ( *A. terreus* ) against *F. oxysporum* suggest the promising role of endophytes as effective biological control agents. Analysis using Gas Chromatography–Mass Spectrometry of the fungal extract detected around 100 compounds (secondary metabolites). In addition to gradually reducing the need for chemical fungicides, bio‐products can also contribute to sustainable agriculture.

## 1 Introduction

Numerous biotic and abiotic stresses influence agriculture, including microbial infections, drought, habitat destruction, soil erosion, temperature fluctuations, nutrient depletion, soil salinity, chemical accumulation from pesticide use, and water loss due to over‐watering (Abdul Rahman, Abdul Hamid, and Nadarajah 2021). Sustainable agricultural practices, such as water management, pollution reduction, and the shift from chemical pesticides to biological controls, are believed to mitigate these challenges (Bale, Van Lenteren, and Bigler 2008). Current research focuses on plant growth promoters that enhance plant growth by providing nutrients or protecting plants from pathogens (biocontrol) (Compant et al. 2005).

Developing a strategy to reduce crop losses before and after harvest and enhance overall crop production is crucial to meet the population's growing demands (Calicioglu et al. 2019). This emphasizes the need to minimize crop yield losses and increase production efficiency. In recent decades, there has been a growing recognition of the risks associated with chemical applications in agriculture, leading to a greater focus on organic farming. Microbial diseases leading to crop losses pose a threat to food security. Certain pathogenic microbes produce metabolites that are harmful to plants, animals, and humans. This growing issue emphasizes the importance of finding and using alternative biological resources for enhancing crop protection and sustainability in agriculture. Utilizing alternative natural resources provides an effective solution to address the increasing demand for food products and the exhaustion of natural resources (Singh et al. 2021). Organic farming practices offer numerous advantages, including ensuring food safety by avoiding harmful chemicals, improving the nutritional quality of food, promoting soil health, and preserving the ecological balance of natural microorganisms. In response to the harmful effects of chemical‐intensive agriculture, the use of plant growth‐promoting microbes as biofertilizers has become more prevalent (Mawar, Manjunatha, and Kumar 2021). A biofertilizer is a live or active microbial inoculant that can be applied to seeds, surfaces of plants, and soils to promote the growth of plants. Several microbes colonize both plant‐internal and plant‐external environments, enabling nutrients to be solubilized and essential metabolites to be produced to support plant growth and health for a long time (Hardoim et al. 2015).

Rhizobacteria and endophytes are believed to play crucial roles in plant life by aiding in nutrient acquisition, such as phosphorus, iron, and nitrogen (Ma et al. 2011; Pattnaik, Mohapatra, and Gupta 2021). Halophytes are plants capable of thriving in saline soils, a habitat considered unsuitable for most other plants, which cannot survive in such conditions (Panta et al. 2014).

The endosphere, a key part of the plant microbiome, is vital for enhancing plant productivity (Dastogeer et al. 2020). The term “endophyte,” coined by De Bary in 1866, merges the Greek words “endon” (meaning within) and “phyton” (meaning plants) to describe a variety of microbes that reside within plant tissues (Petrini 1996). The capacity of endophytes to enhance plant growth highlights their potential as environmentally friendly approaches to increase crop yields (Tiwari, Kang, and Bae 2023). Efforts have been made to enhance the growth and overall well‐being of plants by studying and applying endophytes (Tiwari, Kang, and Bae 2023). The endophytes exhibit numerous advantageous traits for both plants and the environment, playing a significant role in agrosystems (Miliute et al. 2015). Abiotic pressures, such as drought and salinity, and biotic stresses, such as pathogen attacks, have been mitigated with new approaches to agriculture (Chaudhary et al. 2022; Fontana et al. 2021). The close relationship and coevolution between endophytes and their plant hosts have resulted in a variety of benefits for plant growth (Santos et al. 2018). These benefits include enhanced overall plant growth, increased fresh and dry biomass, improved tolerance to biotic and abiotic pressures, and enhanced acquisition and uptake of nutrients and minerals (Mei and Flinn 2010). Some fungal endophytes positively influence plant growth. *Aspergillus nidulans*, an endophytic fungus isolated from *Solanum lycopersicum* , when inoculated in tomato, showed an increased fruit yield and greater overall plant biomass. (Xia et al. 2019). *Stereum gausapatum*, a fungal endophyte, isolated from *Salicornia europaea* has shown growth promotion effects and enhanced salinity tolerance in *Lolium perenne* when inoculated with it (Furtado, Szymańska, and Hrynkiewicz 2019). In the case of bacterial endophytes, the endophytic bacteria *(Pseudomonas spp*. isolates and *Bacillus spp*. isolates) from sugarcane have shown the potential to promote plant growth and enhance host growth under field conditions (Chauhan, Bagyaraj, and Sharma 2013).

Endophytes of halophytes may possess unique properties that contribute to the overall growth and survival of these plants. Studies suggest that these endophytes may play a role in enhancing salt tolerance, nutrient uptake, and overall plant health in halophytes (Etesami and Beattie 2018).

In the Plumbaginaceae family, *Limonium axillare* is a halophytic plant species native to Mediterranean coastal regions and exhibits significant adaptations to aridity and salinity (Abdel‐Bari 2012). Previous studies have focused on isolating bacterial endophytes from *L. axillare* (Alhaddad, Bitaar, and Abu‐Dieyeh 2024). *Bacillus velezensis* strains have shown positive effects in greenhouse experiments, promoting plant growth and showing potential in vitro and in vivo biocontrol against the *Fusarium oxysporum* infection in tomato plants (Alhaddad, Bitaar, and Abu‐Dieyeh 2024).

Tomato crops are susceptible to various fungal pathogens, among which *F. oxysporum* is prominent (Maurya et al. 2019). The symptoms of *F. oxysporum* infection can include yellowing and wilting of the leaves, vascular infection, and stunted growth of the tomato plant (Srinivas et al. 2019). Controlling *Fusarium* and other fungal infections can be achieved with chemicals, but this approach is not environmentally friendly. Therefore, alternative methods need to be optimized, evaluated, and tested (Alberts, van Zyl, and Gelderblom 2016; Heydari and Pessarakli 2010). Intensive research has been evolved to study and evaluate the implementation of plant growth promotor (PGP) for controlling fungal diseases in the plant. For example, *Xylaria feejeensis*, a fungal endophyte isolated from a mangrove tree “(halophyte),” displayed biocontrol activity against *F. oxysporum* in tomato seedling (Brooks et al. 2022). Additionally, the volatile organic compounds produced by the endophytic fungi *Sarocladium brachiariae* HND5, which is isolated from *Brachiaria brizantha* , have shown antifungal activity against *F. oxysporum* f. sp. *cubense* (Yang et al. 2021).

The primary objectives of this study are to isolate endophytic fungi from the leaves of the halophytic plant *L. axillare* at two locations, coastal and inland, to assess colonization rates and frequency. The second objective is to evaluate the isolates' antagonistic potential against four phytopathogens. Lastly, the study aims to inoculate tomato seedlings with the selected endophyte isolate under fungal pathogen (*F. oxysporum*) stress to examine its potential for promoting growth and controlling disease.

## 2 Material and Methods

### 2.1 Sampling and Fungal Endophyte Isolation

The fresh leaves of *L. axillare* (Forssk.) Kuntze were collected from two distinct locations: the inland salt flat area of Qatar University's protected field (Location A) and the coastal sabkha area of Al Thakhira (Location B; ~55 Km apart from A). A total of 30 healthy plants, 15 from each location, were carefully selected for sampling, with each plant spaced at least 1 m apart from the others. This distance was chosen to minimize any potential influence on biodiversity indices due to proximity, ensuring that each plant reflected its unique microenvironment. A total of 30 healthy plants, 15 from each location, were carefully selected for sampling, with each plant spaced 1 m apart from the others. From each plant, eight leaves located 15 cm above the ground were carefully plucked, amounting to 120 leaves per location. These leaves were then promptly placed in zip‐lock labeled plastic bags to ensure proper storage. Subsequently, the bags were transported to the laboratory in an icecontainer to maintain freshness and stored in the lab refrigerator at 4°C. Then, the isolation of endophytes commenced within 24 h from the time of collection to preserve the integrity of the samples. To remove any soil or dust particles, the gathered leaves were thoroughly cleaned with running tap water. This is followed by a surface sterilization process aimed at eliminating any microorganisms, particularly epiphytes, that may be present on the surfaces of leaves. The surface of the leaves sterilization was carried out as follows: The leaves were immersed in a 5% NaClO solution for 2 min, followed by a 5‐min soak in 70% alcohol. Subsequently, the leaves were rinsed several times with sterile dH_2_O to remove any residual sterilizing agents. The leaves were then cut into sections and placed on PDA media plates, which were then incubated at 25°C for up to 30 days to isolate fungi. Fungi emerging from the inner parts of the leaves were subcultured to obtain purified isolates, which were stored at 4°C for later analysis. To confirm the effectiveness of the sterilization process, a sample of the last wash water was covered on PDA media and incubated in the same growing environments. The absence of microbial growth indicated complete sterilization of the samples. The percent rate of colonization was measured by dividing the number of pieces occupied by fungal isolates by the total number of pieces used (Petrini and Carroll 1981).

$$\%\text{rate of colonization}=\left(\text{number of leaf fragments colonized}\;\mathrm{by}\;\text{fungi}/\text{total number of leaf fragments}\right)*100\%.$$

For each endophytic fungi species, the colonization frequency percent (CF) was calculated according to the following formula (Hata and Futai 1995; Jinu and Jayabaskaran 2015; Sun et al. 2011):

$$\mathrm{CF}=\frac{\text{Ncol}}{\mathrm{Nt}}\times 100,$$

where *N*_*col*_ represents the number of leaf segments colonized by each fungal species and *N*_*t*_ represents the total number of examined leaves segments. Diversity was measured by the Shannon‐Wiener Diversity Index (*H*′) (Shannon 1948):

$$H^{\prime }=-\sum_{i=1}^{n}\mathrm{pi}\ln\mathrm{pi},$$

where *p*_*i*_ refers to the proportion of *i* ^th^ species, *ln* the natural logarithm of *p*_*i*_, and *n* refers to the number of individual of particular species. Simpson's index for diversity was measured according to the following formula (Simpson 1949):

$$D=1-\frac{\sum n\left(n-1\right)}{N\left(N-1\right)},$$

where *N* refers to the total number of individual organisms and *n* refers to the number of individuals of a particular species. Species Evenness was measured by Pielou's Evenness (*J*) (Pielou 1966):

$$J=\frac{H^{\prime }}{\ln\left(s\right)}.$$

Therein, *H*′ is the value of the Shannon‐Wiener index, and ln (*s*) is the natural log of the species richness (the total number of different species). Similarity among the endophytic species that were isolated from the leaves of two different locations was measured according to Sorensen's similarity index (SI) (Magurran 1988):

$$\mathrm{SI}=\frac{2C}{A+B},$$

where *C* refers to the common species in both locations and *A* and *B* refer to the different species in each location. Menhinick's richness index (*D*_*mn*_) (Menhinick 1964) was calculated as

$$D_{\mathrm{mn}}=\frac{s}{\surd N},$$

where *s* is the number of different species and *N* is the total number of isolates.

### 2.2 Molecular Ribotyping for Fungal Endophytes Identification

The endophytic fungi were molecularly identified through (1) the extraction of total genomic DNA, (2) PCR procedures, (3) sequencing of nucleotide, and (4) strains and phylogenetic analysis. The total genomic DNA of each fungal isolate was extracted from one weak old fresh mycelia by using DNeasy PowerSoil Kit (Qiagen GmbH, Hilden, Germany) as per the company procedure. To identify the isolates, the Internal Transcribed Spacer (ITS) region of the ribosomal DNA was sequenced by PCR using the universal primer pair (ITS1: [TCCGTTGGTGAACCAGCGG] and ITS4: [TCCTCCGCTTATTGATATGC]) (White et al. 1990). The sequencing of the PCR products was performed using the Sanger method, followed by using BioEdit software (https://bioedit.software.informer.com/) to read the sequences. The amplified sequences of endophytes were compared with available sequences in the NCBI‐BLAST for species identification (Raja et al. 2017). Using MEGA Version 11 software (https://www.megasoftware.net/), a phylogenetic tree was created employing the Maximum Likelihood method.

### 2.3 Antagonistic Effect of Endophytic Fungal Isolates Against Fungal Phytopathogens

An antagonistic interaction between fungi and plant pathogens was evaluated using the dual culture assay of isolated endophytes against plant pathogenic fungi: *Alternaria alternate*, *Colletotrichum gloeosporioides*, *Botrytis cinerea*, and *F. oxysporum* (Trejo‐Estrada, Sepulveda, and Crawford 1998). In this assay, a 4‐mm‐diameter disk of fresh mycelium growth from 1‐week‐old cultures was placed 2 cm away from the edge of a 90‐mm PDA plate. The experimental plates contained the phytopathogen and the *fungal endophyte* isolate placed at opposite edges, whereas the control plates contained only the phytopathogen disk located 2 cm from the plate border. The PDA plates were then incubated at 24 ± 2°C for 10 days. Four replicates of each treatment were conducted, and the obtained outcomes were expressed as means of the replicates ± standard deviation (SD). The percentage of pathogenic growth inhibition was calculated using the formula: percentage Inhibition = [(*C* − *T*)/*C*] × 100, where *T* is the radius of the fungal mycelia treated with the endophyte and *C* is the radius of the fungal mycelia in the control plate.

### 2.4 Bioactive Compounds of Selected Fungi Extraction and Gas Chromatography–Mass Spectrometry (GC‐MS) Analysis

The extraction of bioactive compounds from fungi was performed by preparing the filtrate from the fungal culture broth (Wang et al. 2007). Briefly, fungi were introduced into a 250‐mL flask containing 150 mL of purified broth (PD) media and placed in a shaker incubator at 25 ± 2°C, rotating at 130 rpm for 2 weeks. After incubation, the media were filtered by first centrifuging the culture broth to collect the supernatant. The supernatant was then filtered using a syringe with a 0.45‐μm pore size syringe filter. The filters were then submitted to the ESC (Environmental Science Center) at Qatar University for performing extraction of the bioactive compounds and applying the extracted solution to GC‐MS for scan mode analysis as described in our previous paper (Alhaddad et al. 2024).

### 2.5 Greenhouse Experiment for Bioassay

Tomato seeds ( *Solanum lycopersicum* L., Marglobe variety from Italy), purchased from a local market, were surface‐sterilized to ensure sterility for the experiment. This process involved dipping the seeds in 1% sodium hypochlorite for 1 min, followed by 70% ethanol for 3 min, and finally washing them several times with distilled water to remove any residue from the sterilization agents. One‐week‐old seedlings were then transplanted into 10‐cm pots and inoculated with a soil‐to‐peat moss mixture (1:2 ratio). The experiment was conducted in a greenhouse, with one variable tested across four treatment levels and four replicates. The experiment was repeated twice, yielding consistent results. The treatments were initiated on 2‐week‐old tomato seedlings as follows:

1. Control.
2. Endophyte treatment (AL10).
3. *F. oxysporum* treatment.
4. Endophyte (AL10) and *F. oxysporum* treatment.

The assigned treatments began when the seedlings reached 2 weeks old for endophytic inoculation and 3 weeks old for treatments with fungal inoculation as outlined below:

- Control treatments: Seedlings received no treatment.
- Treatments 2 and 4: Seedlings were inoculated with approximately 10^8^ conidial of the endophytic fungal suspension from the isolate (AL10).
- Treatment 3: Seedlings were inoculated with approximately 10^8^ conidial of *F. oxysporum* suspension. A preliminary investigation in a growth chamber experiment indicated that the fungus *F. oxysporum* caused infection to the tested cultivar of tomato seedlings.
- Treatment 4 involved applying the fungal pathogen suspension 1 week after the endophytic inoculation to ensure the endophytes colonized the seedlings before initiating the fungal disease. The potential of the endophyte to colonize the root of tomato seedlings were preliminary investigated in a growth chamber experiment, and we were able to isolate the same endophyte from tomato seedling roots.

The experiment spanned a 2‐month duration, during which various growth parameters were meticulously recorded for subsequent analysis. These parameters included shoot height, the number of branches, leaf chlorophyll contents, and fresh and dry weights for both aboveground and belowground components. Chlorophyll content was measured using a SPAD 502 (Plus) Chlorophyll meter (Konica Minolta, Inc.). To weigh the seedlings, they were separated into aboveground and belowground portions, and their fresh weights were recorded. Subsequently, the seedling parts were dried in an oven at 80°C for 4 days, after which their dry weights were recorded for both aboveground and belowground portions.

### 2.6 Statistical Analysis

OriginPro (Version 2023; OriginLab Corporation, United States) was used for statistical analysis, including one‐way analysis of variance (ANOVA) and Tukey's pairwise comparisons to compare the means at *p* ≤ 0.05.

## 3 Results

### 3.1 Identification and Biodiversity of Fungal Endophytes

**FIGURE 1:**
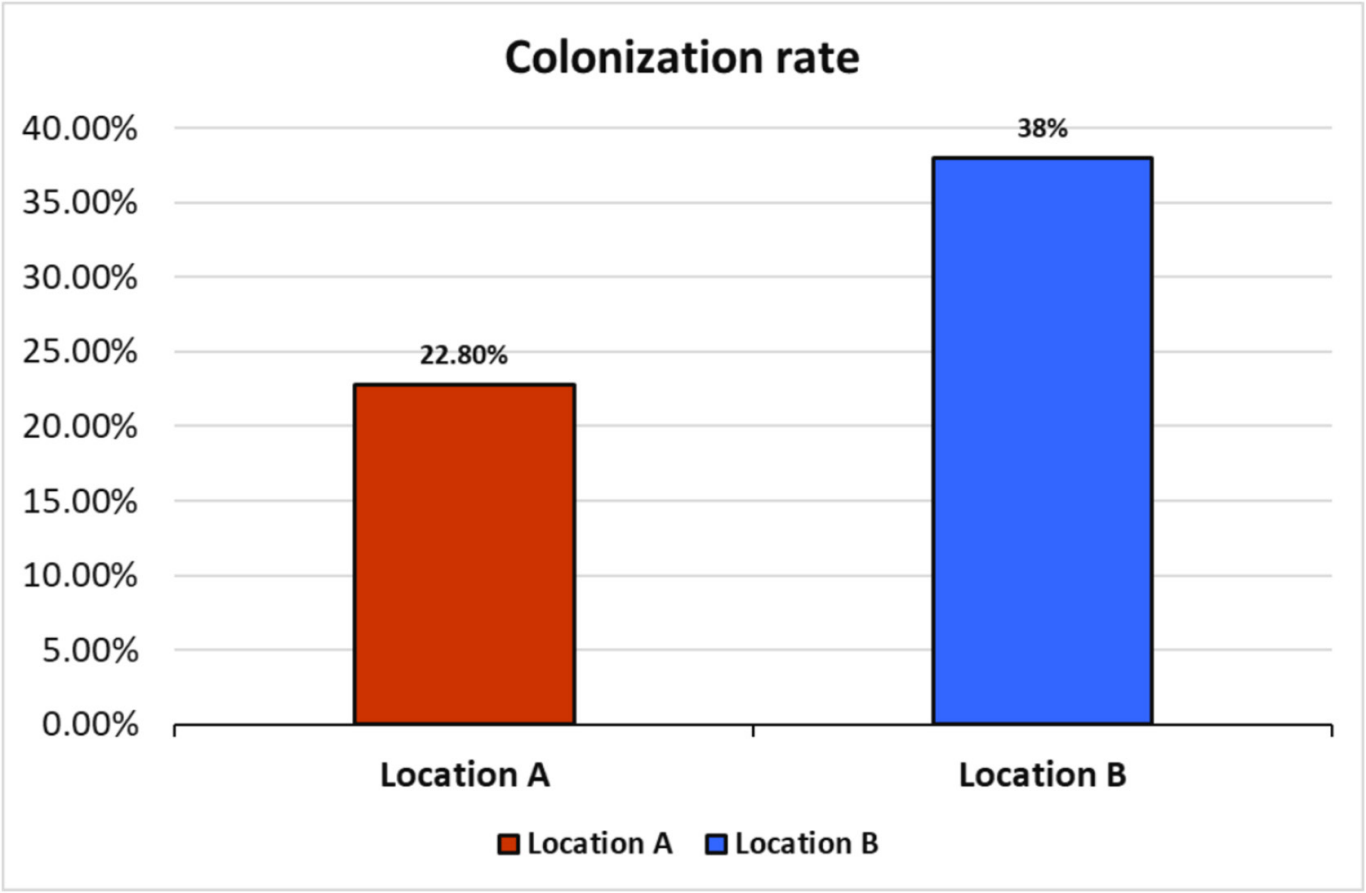
Colonization rate for endophytic fungi isolated from *Limonium axillare* of two different locations: Location A inland and Location B coastal habitat.

**FIGURE 2:**
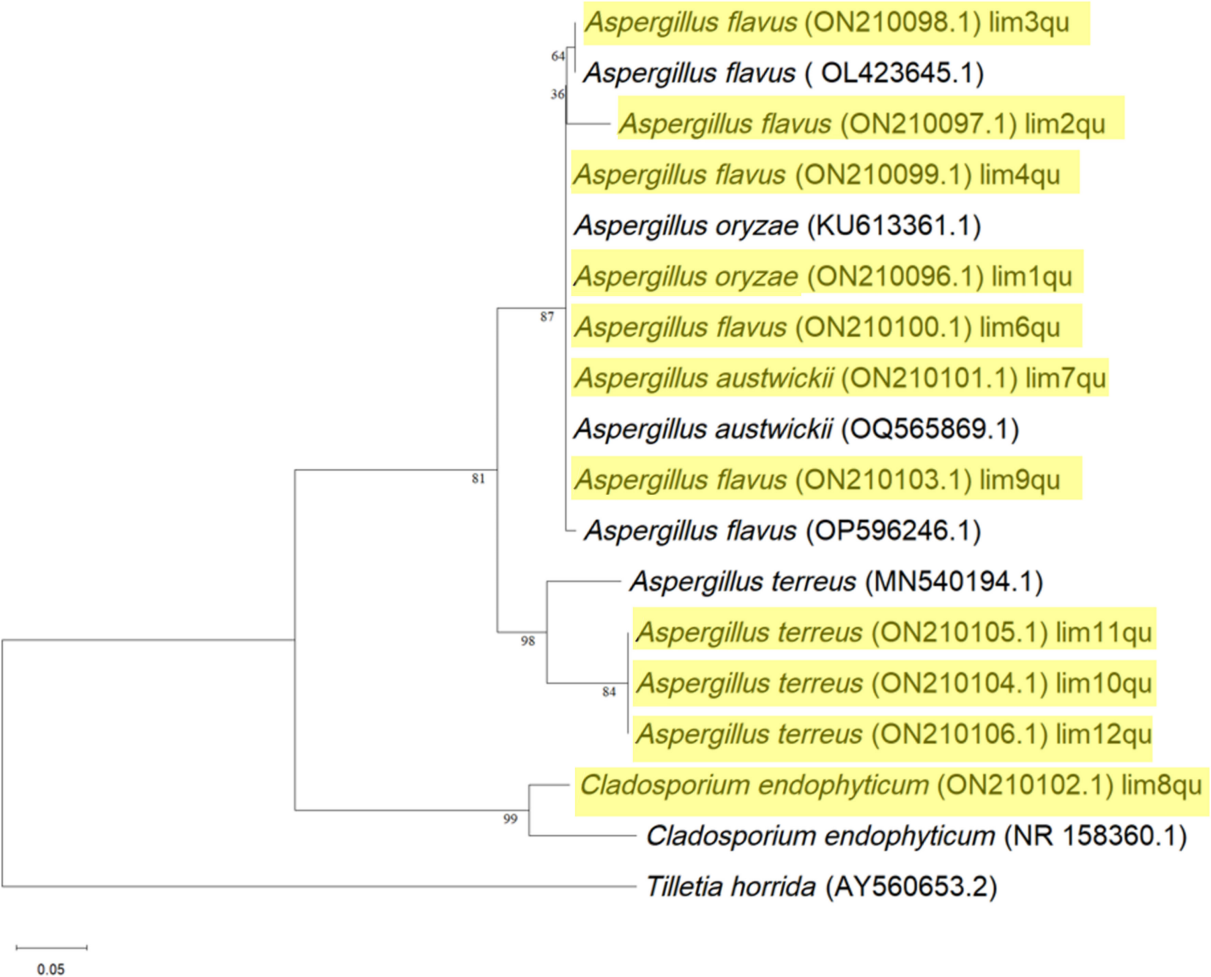
Phylogenetic analysis conducted by MEGA 11, using the Maximum Likelihood method of 1000 bootstrap comparisons and Kimura two‐parameter model for the 11 (highlighted) taxa of endophytic fungi isolated from *Limonium axillare* based on ITS region. *Tilletia horrida* (AY560653.2) is the outgroup.

Endophytic fungi were isolated from the leaves of *L. axillare*, a halophytic plant, collected from two main locations: Location A (the inland area of Qatar University's protected field) and Location B (the coastal area of Al Thakhira). A total of 120 leaves from each location were analyzed in this study, with 250 leaf segments from each sampling location examined for the emergence of fungal endophytes. The overall number of isolated endophytic fungi from both locations was 152 isolates, comprising 95 isolates from the coastal plants and 57 isolates from the inland plants samples. The colonization rate of isolates from the coastal area (Location B) was 38% higher than that of inland isolates (Location A), which was 22.8% (Figure 1). All collected isolates were categorized into 11 taxa based on morphological characterization. One isolate from each taxa underwent molecular identification, and plant‐fungi pathogen antagonistic tests, and the strain that showed the maximum reduction in the mycelium growth of the phytopathogen underwent plant bioassay to test its potential biocontrol activity. The extracted total genomic DNA was analyzed using the ITS region of the rRNA gene. The molecular characterization of the fungi, rDNA‐ITS region, was amplified by PCR with primers pair ITS1 (forward) and ITS4 (reverse). Fragment sizes of about 500–600 bp were obtained. The PCR products were sequenced and aligned to other sequences in the NCBI‐BLASTn database. The obtained sequences were subsequently deposited into the GenBank database, where they were assigned unique accession numbers for each fungal endophytic isolate (Table 1). The results revealed that all the endophytic isolates belonged to the phylum Ascomycota in which approximately 90% of the isolates were identified as belonging to the genus *Aspergillus* including four different species (*A. austwickii*, *A.oryzae*, *A. flavus* , and *A. terreus* ). Additionally, one taxon belonged to the genus *Cladosporium*, encompassing the species *Cladosporium endophyticum* as we can bserve in the phylogenetic tree (Figure 2).

**TABLE 1:** Molecular identification for fungal endophyte isolates by 16S rDNA sequencing.

No.	Morphological identification	Name of the fungi identified	Isolate code	NCBI accession number	Name of the isolates matching with NCBI database and their accession numbers	% identity
1	*Aspergillus* sp.	*Aspergillus oryzae*	(AL1) lim1qu	ON210096.1	*Aspergillus oryzae* (KU613361.1)	100%
2	*Aspergillus* sp.	*Aspergillus flavus*	(AL2) lim2qu	ON210097.1	*Aspergillus flavus* (MZ723884.1)	98%
3	*Aspergillus* sp.	*Aspergillus flavus*	(AL3) lim3qu	ON210098.1	*Aspergillus flavus* (MT584285.1)	100%
4	*Aspergillus* sp.	*Aspergillus flavus*	(AL4) lim4qu	ON210099.1	*Aspergillus flavus* (ON981297.1)	100%
5	*Aspergillus* sp.	*Aspergillus flavus*	(AL6) lim6qu	ON210100.1	*Aspergillus flavus* (OQ506578.1)	99%
6	*Aspergillus* sp.	*Aspergillus austwickii*	(AL7) lim7qu	ON210101.1	*Aspergillus austwickii* (ON332136.1)	99%
7	*Cladosporium* sp.	*Cladosporium endophyticum*	(AL8) lim8qu	ON210102.1	*Cladosporium endophyticum* (MN577241.1)	100%
8	*Aspergillus* sp.	*Aspergillus flavus*	(AL9) lim9qu	ON210103.1	*Aspergillus flavus* (OQ874799.1)	100%
9	*Aspergillus* sp.	*Aspergillus terreus*	(AL10) lim10qu	ON210104.1	*Aspergillus terreus* (ON358398.1)	99%
10	*Aspergillus* sp.	*Aspergillus terreus*	(AL11) lim11qu	ON210105.1	*Aspergillus terreus* (OQ519782.1)	99%
11	*Aspergillus* sp.	*Aspergillus terreus*	(AL12) lim12qu	ON210106.1	*Aspergillus terreus* (MN540194.1)	99%

**FIGURE 3:**
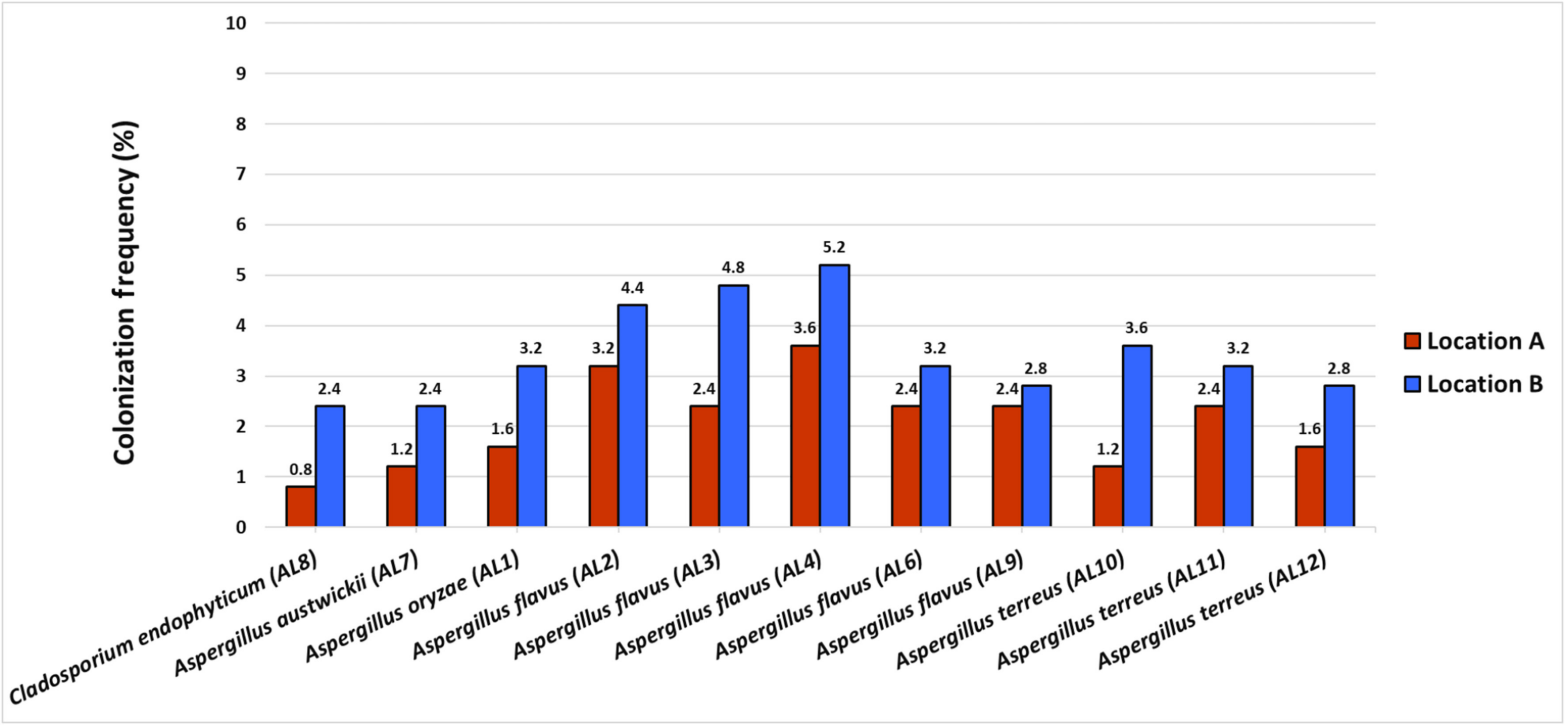
Colonization frequency (CF) percentage of fungal endophytes from the leaves of the halophytic plant *L. axillare.*

**FIGURE 4:**
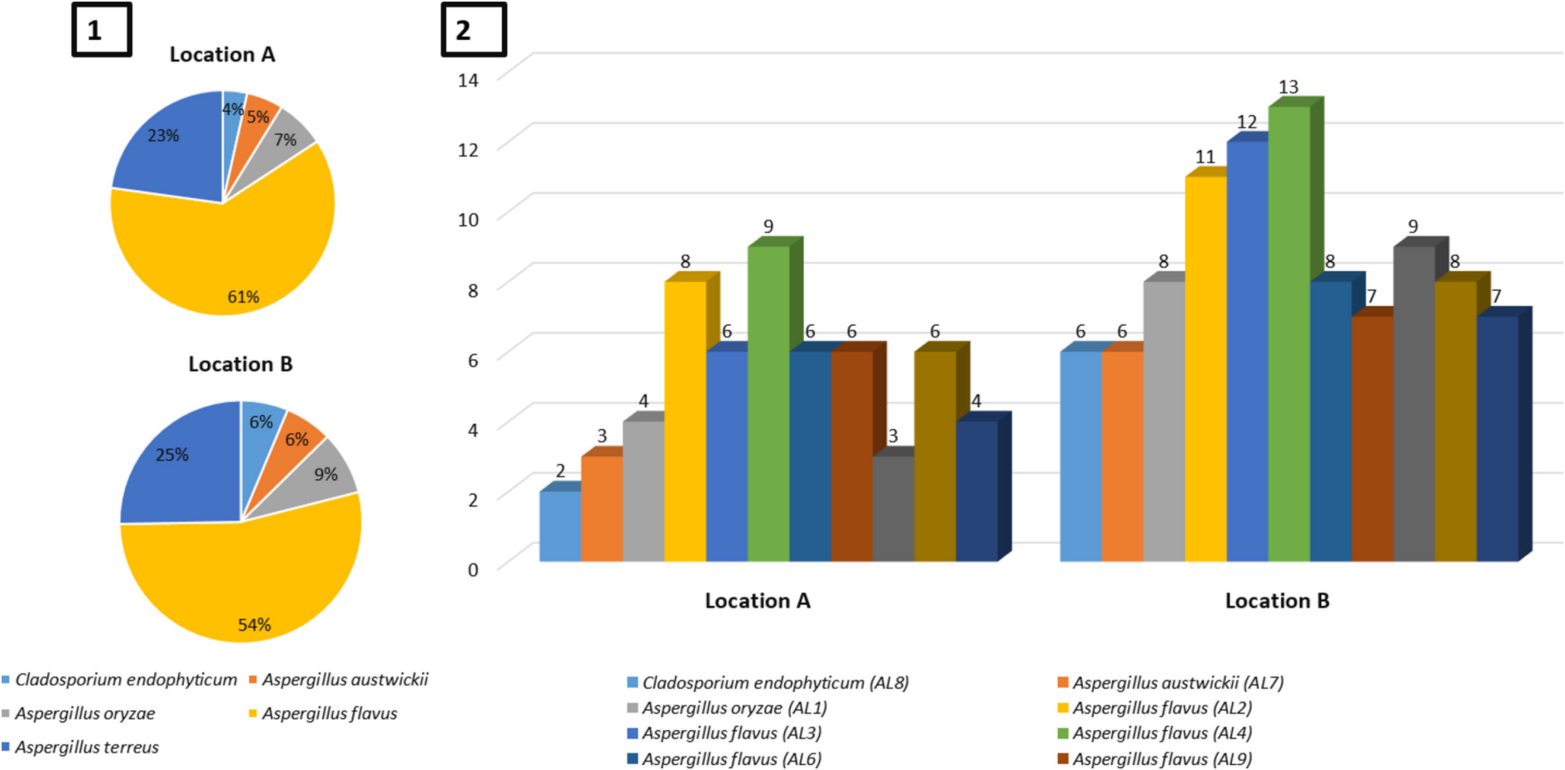
Comparison of fungal endophytic species abundance isolated from *L. axillare* at two distinct locations: Location A (inland habitat) and Location B (coastal habitat). The abundance is presented based on (1) species level and (2) identified taxa.

Both locations showed similarities in terms of species richness with variations in colonization frequency among different taxa (Figure 3). The overall colonization frequency was higher for Location B compared to Location A. The species *Aspergillus flavus* (AL4) exhibited the highest colonization frequency in both locations, across all analyzed leaf segments. The abundance of fungal endophytic species isolated from *L. axillare* was higher in Location B. At the species level, *A. flavus* showed the highest abundance in both locations, followed by *A. terreus*, *Aspergillus oryzae*, *Aspergillus austwickii*, and *C. endophyticum* (Figure 4).

The diversity indices of endophytic fungal species isolated from leaf tissues of *L. axillare* of two locations are listed in Table 2. In this table, Location B has a higher Shannon–Wiener index (1.23) compared to Location A (1.10), suggesting that the fungal species diversity is higher at Location B. Simpson's diversity (1‐D) index is complementary to the Shannon–Wiener index and also measures species diversity: The more the value closer to 1 means the more diverse the population is. In other words, higher values indicate higher diversity. Location B has a higher Simpson's diversity value (0.64) compared to Location A (0.57), which again suggests higher diversity at Location. The Evenness (J) index indicates how evenly the species are distributed in a community. A value of 1 indicates perfect evenness. Both locations have high evenness values (0.683 for Location A and 0.746 for Location B), suggesting a relatively even distribution of fungal species in both habitats. Both Locations A and B have the same species richness (five species each). Meinhinick's richness index (Dmn) estimates the number of species in a community based on the abundance of individuals. A higher index indicates higher species richness. Location A has a higher richness index (0.66) compared to Location B (0.51), indicating a higher estimated number of species in Location A. Sorenson's similarity (SI) index measures the similarity between two communities in terms of species composition. A value of 1 indicates identical species composition. In this case, Sorenson's similarity index between Locations A and B is 1, suggesting that the two locations have identical species compositions despite differences in diversity and richness indices.

**TABLE 2:** Diversity of endophyic fungi isolated from leaves of *L. axillare* at two distinct locations: Location A (inland habitat) and Location B (coastal habitat).

	Shannon‐Wiener index *H*′	Evenness *J*	Simpson's diversity index (1‐D)	Menhinick's richness index (*D*_*mn*_)	Species richness	Sorensen's similarity *SI*
Location A	1.10	0.683	0.57	0.66	5	1
Location B	1.23	0.746	0.64	0.51	5	

### 3.2 In Vitro Antagonistic Activities of Fungal Endophytes Against Plant Pathogens

The fungicidal effect of the fungal endophytic isolates varies from one isolate to another when tested against different fungal phytopathogens (Table 3). In dual culture with four phytopathogens, the isolates were evaluated for their antifungal efficacy. All findings were compared with the growth of the corresponding phytopathogens on control plates. The fungal isolate (AL10) has shown a maximum reduction in the *F. oxysporum* mycelium growth. Due to this significant antifungal activity, it was selected for further analysis of its secondary metabolites and evaluated for its potential as a biocontrol agent against fungal diseases under greenhouse conditions.

**TABLE 3:** Antagonistic properties of fungal endophytes towards fungal phytopathogens (mean ± S.D).

Fungal endophytes	Isolate code	% growth inhibition of fungal pathogens			
		*A. alternata*	*B. cinerea*	*F. oxysporum*	*C. gloeosporioides*
*A.oryzae*	(AL1) lim1qu	37.1 ± 0.7	64.9 ± 1.4	00.0 ± 0.0	50.6 ± 1.0
*A. flavus*	(AL2) lim2qu	44.4 ± 1.6	10.3 ± 0.6	24.5 ± 0.6	63.3 ± 2.4
*A. flavus*	(AL3) lim3qu	12.0 ± 0.8	21.3 ± 1.5	57.5 ± 0.3	44.5 ± 0.4
*A. flavus*	(AL4) lim4qu	62.5 ± 1.5	46.4 ± 1.4	24.5 ± 0.6	55.2 ± 0.0
*A. flavus*	(AL6) lim6qu	24.5 ± 0.6	63.0 ± 1.2	37.1 ± 0.7	19.6 ± 0.7
*A. austwickii*	(AL7) lim7qu	57.1 ± 1.4	14.5 ± 1.5	05.0 ± 2.3	44.5 ± 0.4
*C. endophyticum*	(AL8) lim8qu	42.5 ± 0.2	54.8 ± 0.6	24.5 ± 0.6	37.4 ± 0.6
*A. flavus*	(AL9) lim9qu	30.4 ± 0.4	44.5 ± 0.4	54.9 ± 1.4	67.0 ± 0.7
*A. terreus*	(AL10) lim10qu	47.1 ± 0.7	72.8 ± 1.6	71.4 ± 0.9	57.7 ± 0.5
*A. terreus*	(AL11) lim11qu	63.5 ± 0.1	17.4 ± 0.2	43.2 ± 1.9	20.4 ± 1.2
*A. terreus*	(AL12) lim12qu	49.8 ± 0.4	13.9 ± 1.3	30.4 ± 1.3	53.6 ± 0.5

### 3.3 Screening for the Secondary Metabolites of Fungal Isolate (AL10) Analyzed by GC‐MS

**FIGURE 5:**
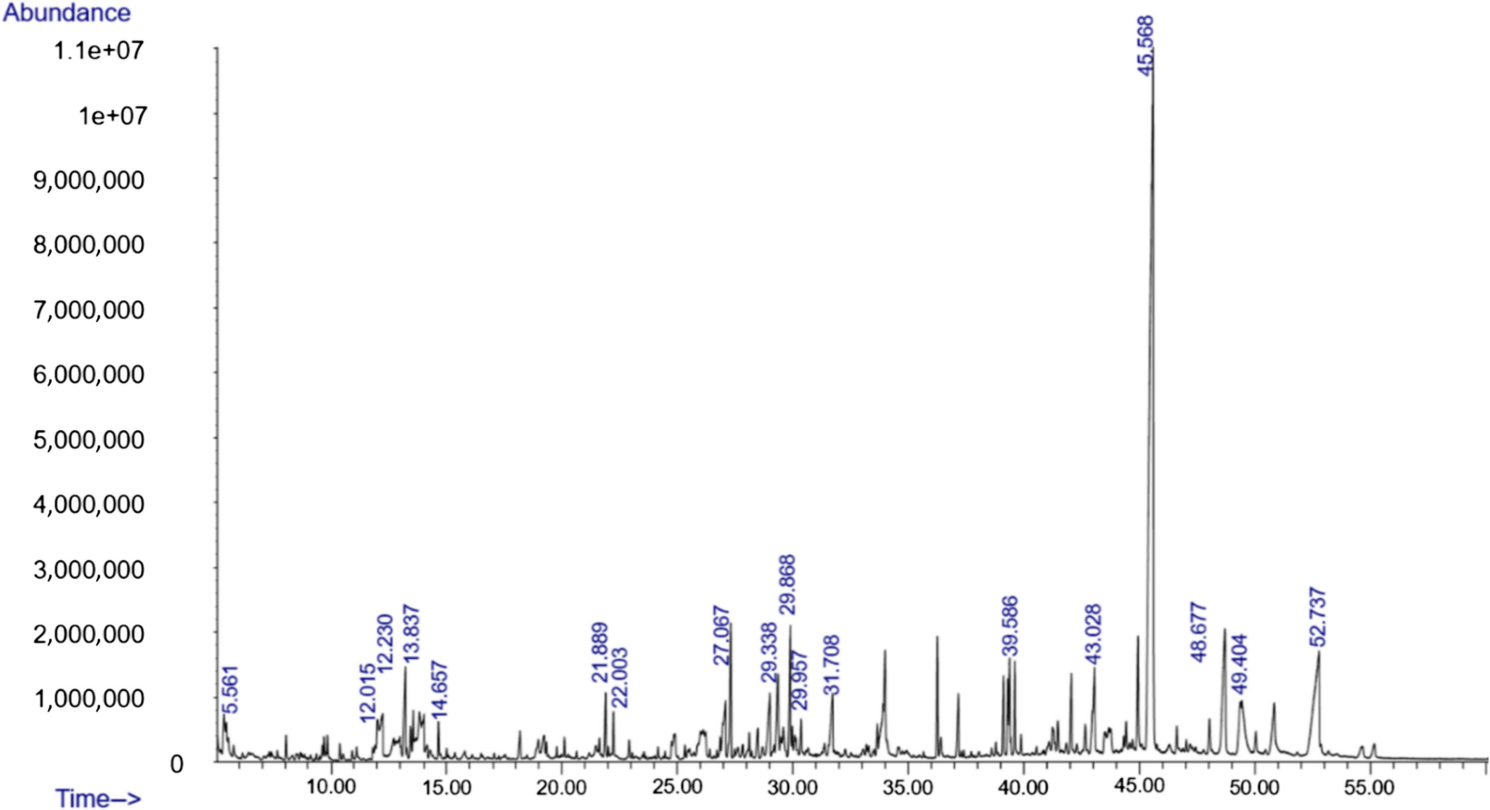
GC‐MS scan mode chromatogram of extract of the endophyte AL10.

Fungi use secondary metabolites to enhance their growth and bolster defenses against pathogens. Fungal endophytes, specifically, are vital in aiding plants either directly or indirectly by promoting growth and offering biocontrol against plant pathogens. Understanding the functional roles of these metabolites and discovering compounds with potential benefits are crucial steps in this process. The GC‐MS revealed the presence of about 100 compounds, as shown in Figure 5. The major compounds present in dichloromethane extraction of the endophytic fungi AL10 are represented in Table 4. Certain primary components that were present in the culture filtrate of the endophytic fungi *A. terreus* (AL10) have been documented as medicinal constituents in the previous studies. For example, 2‐phenylacetic acid is recognized for its potent antimicrobial and antityrosinase properties (Zhu et al. 2011). 3,5‐Dihydroxybenzoic acid, a type of hydroxybenzoic acid, can act as an antioxidant and have other metabolic roles in plants (Kalinowska et al. 2021).

**TABLE 4:** The primary bioactive compounds in the dichloromethane extract of fungal endophyte AL10 were analyzed through GC/MS scan mode.

Peak No.	Retention time	Peak area %	Name of the compound	Molecular formula	Molecular weight (g/mol)	Structure
1	5.56	0.26	Hept‐1‐ene	C_7_ H_14_	98.19	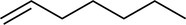
2	12.02	1	2,3‐Dihydrothiophene	C_4_ H_6_ S	86.16	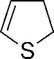
3	12.23	1.22	Pyrrolidin‐1‐amine	C_4_ H_10_ N_2_	86.14	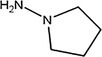
4	13.84	1.44	2‐Phenylacetic acid	C_8_ H_8_ O_2_	136.15	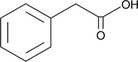
5	14.66	0.36	4‐Tert‐butylphenol	C_10_ H_14_ O	150.22	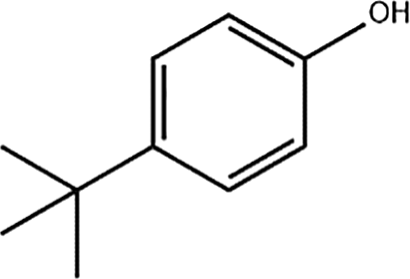
6	21.89	0.79	Cyclohexanepropanal—	C_9_ H_16_ O	140.22	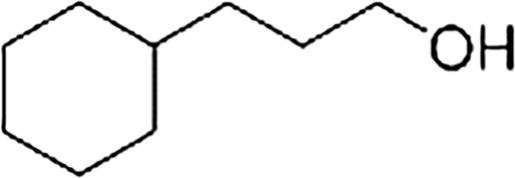
7	22.00	0.16	Isoxanthopterin	C_6_ H_5_ N_5_ O_2_	179.14	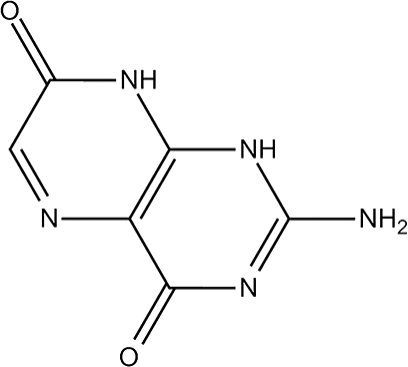
8	27.07	1.56	3,5‐Dihydroxybenzoic acid	C_7_ H_6_ O_4_	154.12	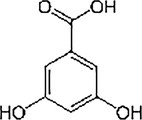
9	29.34	1.63	1, 4‐Diaza‐2, 5‐dioxo‐3‐isobutyl bicyclo[4.3.0]nonane	C_11_ H_18_ N_2_ O_2_	210	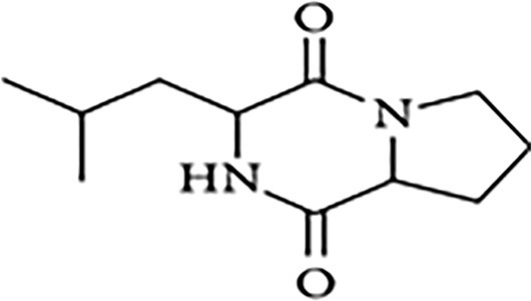
10	29.87	1.77	3,3‐Dimethyl‐2‐methylenenorbornane	C_10_ H_16_	136.24	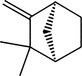
11	29.96	0.35	Hexadecanoic acid	C_16_ H_32_ O_2_	256.42	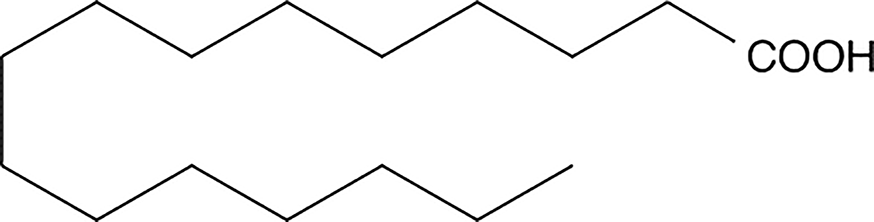
12	31.71	1.49	(1S,4aR,8aR)‐7‐Methyl‐4‐methylidene‐1‐propan‐2‐yl‐2,3,4a,5,6,8a‐hexahydro‐1H‐naphthalene	C_15_ H_24_	204.35	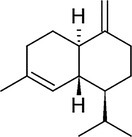
13	39.59	1.03	3‐Nitrophthalic acid	C_8_ H_5_ NO_6_	211.13	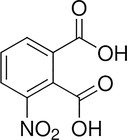
14	43.03	1.97	Trimethyl(2‐phenylethynyl)silane	C_11_ H_14_ Si	174.31	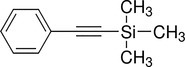
15	45.57	25.91	α‐Ionene (IUPAC name: 1,1,6‐Trimethyl‐1,2,3,4‐tetrahydronaphthalen)	C_13_ H_18_	174.28	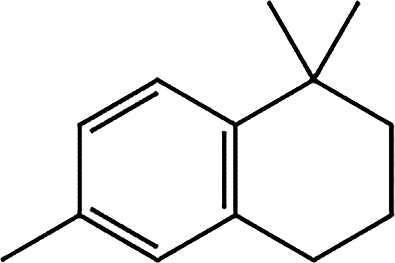
16	48.68	3.75	Lovastatin	C_24_ H_36_ O_5_	404.5	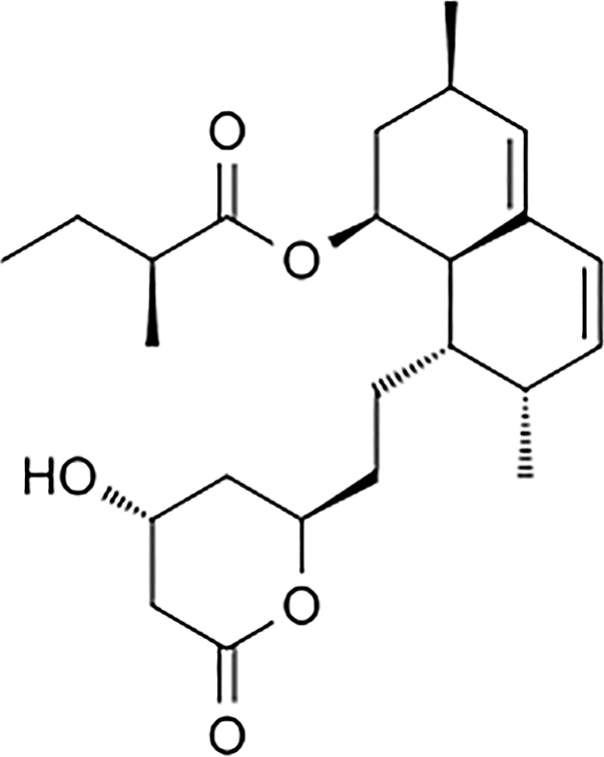
17	49.40	3.27	4‐(4‐methoxyphenyl)‐2H‐triazole	C_9_ H_9_ N_3_ O	175.19	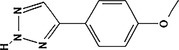
18	52.74	6.22	1,4‐Dimethyl‐2,5‐di (propan‐2‐yl)benzene	C_14_ H_22_	190.32	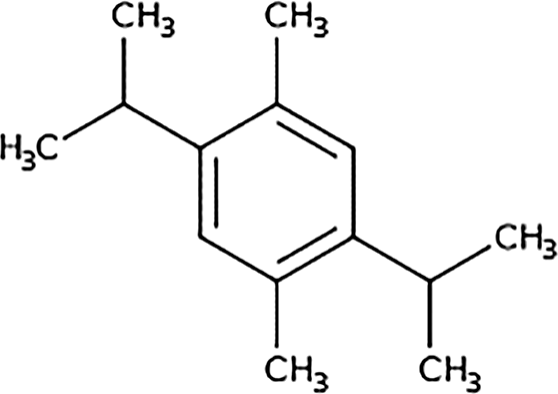

### 3.4 The Biocontrol and Growth‐Promoting Effects of the Endophytic Isolate Strain ([AL10] lim10qu) (ON210104.1) ON Tomato Seedlings

Antagonistic potential was assessed through dual‐culture assays against phytopathogenic fungi. The isolates exhibited varying degrees of antagonistic activity against phytopathogens, highlighting their potential role in plant protection. Further studies are necessary to elucidate the mechanisms underlying these interactions and to explore their practical applications in agriculture. An endophytic isolate of *A. terreus* strain ([AL10] lim10qu) (ON210104.1) exhibited potent in vitro antifungal activity against the plant pathogen *F. oxysporum*, a fungus known for its pathogenic effects on tomato plants. Four treatments were studied: ([1] control) seedlings without treatment, ([2] fungal endophyte AL10) seedlings infected with fungal endophyte (AL10), ([3] pathogenic fungi *F. oxysporum*) seedlings infected with the spore suspension of pathogen *F.oxysporum*, and ([4] endophyte AL10 + *F. oxysporum*) seedlings inoculated first with endophyte spore suspension (AL10) and then with the spore suspension of the plant pathogen *F. oxysporum*.

**FIGURE 6:**
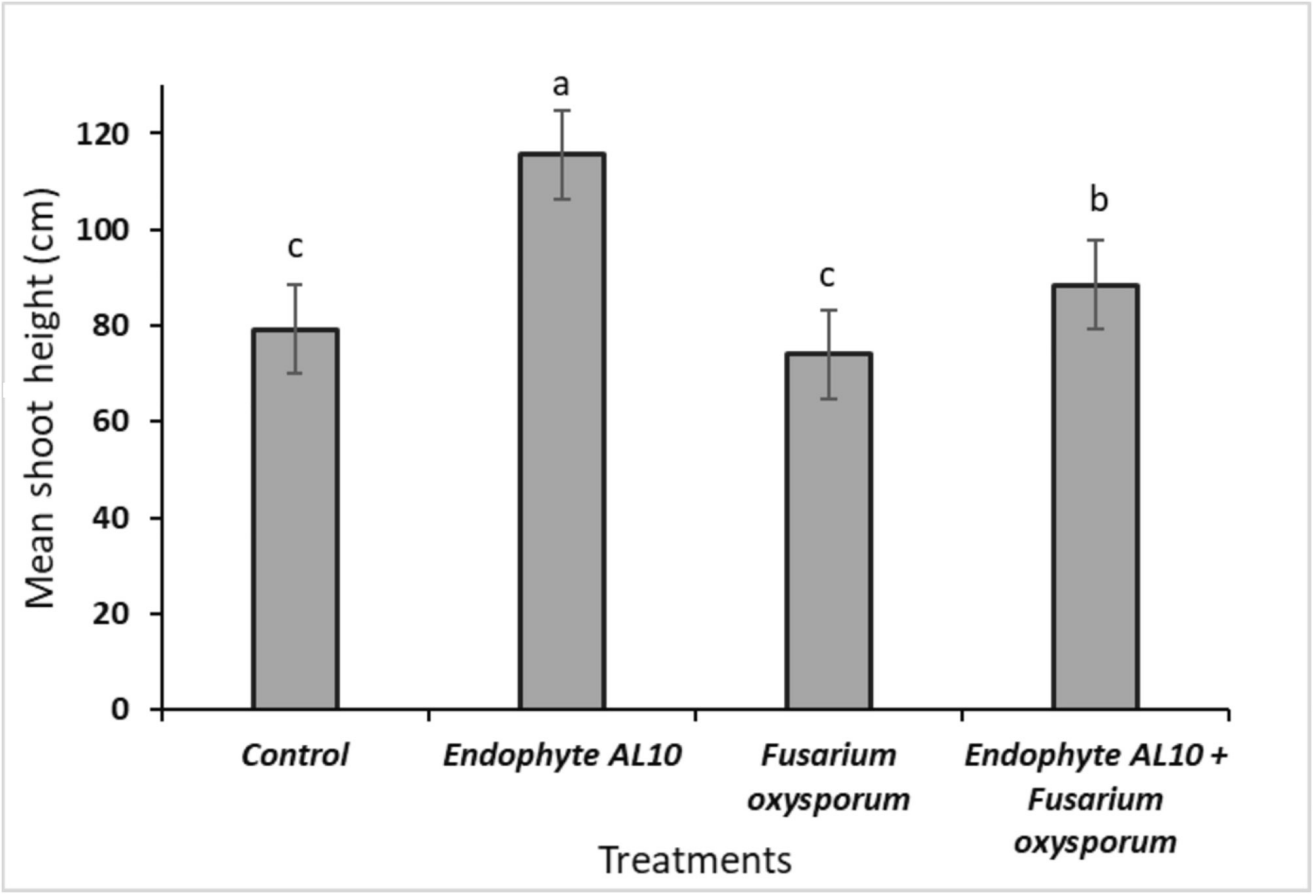
Impact of fungal endophyte (AL10) and pathogen *F. oxysporum* treatments on the shoot height of tomato seedlings after 8 weeks (*N* = 4). Error bars represent standard errors of the means. Values with the same letter(s) are considered statistically not significant at *p* ≤ 0.05 according to Tukey's test.

**FIGURE 7:**
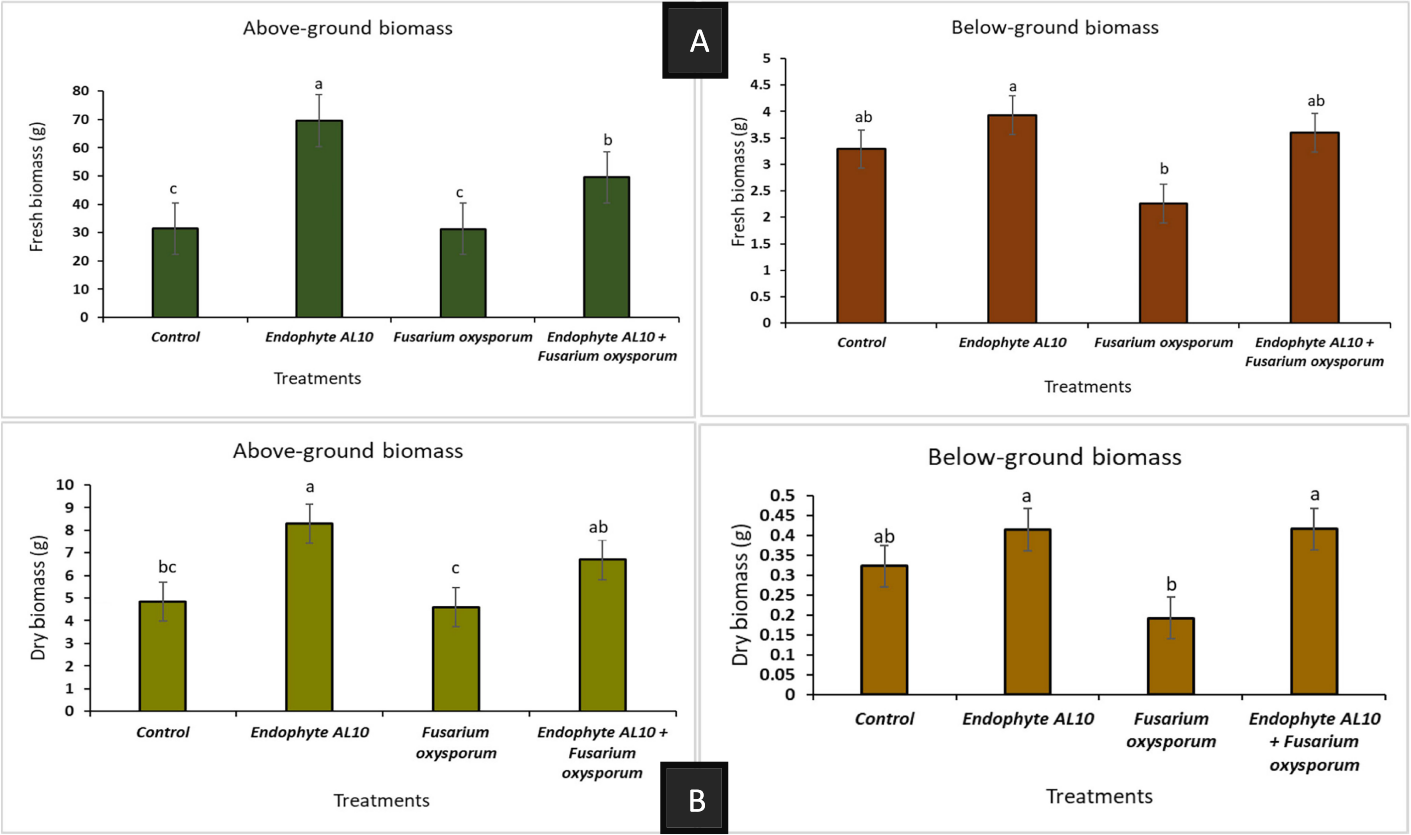
Impact of endophyte and pathogen treatments on the biomass of tomato seedlings, including both aboveground and belowground biomass, after 8 weeks (*N* = 4). Graph (A) shows the fresh weight biomass for aboveground and belowground parts, whereas Graph (B) depicts the dry weight biomass for these sections. Error bars indicate standard errors of the means. Values sharing the same letter(s) are deemed not significant at *p* ≤ 0.05 according to Tukey's test.

**FIGURE 8:**
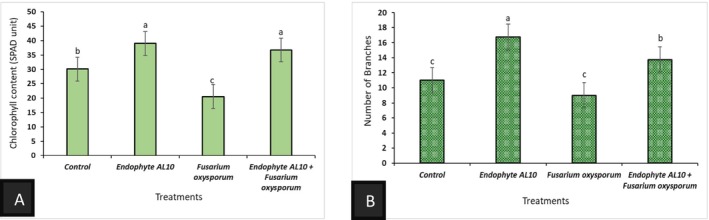
Graph (A) represents the chlorophyll content in the tomato under different treatments. Graph (B) represents the number of branches of the tomato seedlings under different treatments. Error bars represent the standard errors of the means. Values with the same letter(s) are considered not significantly different at *p* ≤ 0.05 according to Tukey's test.

The growth parameters were recorded and graphically presented, including shoot height, fresh, and dry biomass weights for both above and below‐ground, chlorophyll content, and number of branches. Greenhouse experiments demonstrated that the endophytic fungi (AL10) significantly increased the length of tomato seedlings, the chlorophyll content, number of branches, and the overall plant biomass (Figures 6, 7, 8).

## 4 Discussion

The global food supply faces a significant threat to food security from multiple factors, including crop losses caused by microbial diseases, pest and insect attacks, poor agricultural practices, climate change, and water scarcity (Savary et al. 2012; Strange and Scott 2005). Therefore, there is a call for innovation to improve the quality and quantity of food production to maintain a sustainable supply. One of these innovations is the utilization of plant growth‐promoting microorganisms (PGP). In this study, we examined and analyzed the isolation and identification of endophytic fungi from a halophytic plant in two locations (inland and coastal). These isolates could help their host plant withstand salinity, heat, and drought and defend against various phytopathogens (Singh et al. 2021; Verma et al. 2021; White et al. 2019). In a previous study, the endophytic bacteria were extracted from surface sterilized leaves of *L. axillare*, identified, and analyzed for growth promotor (Alhaddad, Bitaar, and Abu‐Dieyeh 2024). There have been several studies on endophytic fungi from the *Limonium* genus, such as *L. aureum* (Liu et al. 2013), *L. tubiflorum* (Aly et al. 2011), and *L. tetragonum* (Khalmuratova et al. 2021; Khalmuratova et al. 2020). However, to our knowledge, this is the first research on isolating, characterizing, and studying of endophytic fungi from the species *L. axillare*. These fungi were then applied in vivo to enhance the growth performance of tomato plants.

This current study focused on isolating fungal endophytes only from *L. axillare* from two distinct coastal and inland locations. A total of 152 isolates were successfully obtained after surface sterilization of leaves, with 95 isolates originating from coastal plants and 57 isolates from inland plants. The obtained strains were classified into 11 taxas. Eleven strains from each taxon were identified up to the species level by ITS sequences and deposited in the NCBI. In China, endophytic fungi were isolated from the medicinal plant *L. aureum* (Liu et al. 2013), receiving a total of 21 different endophytic fungi. Thirty‐nine isolates were obtained from *L. tetragonum* and classified into 13 genera and 17 species (Khalmuratova et al. 2021).

The frequency of colonization data indicates that various endophytic fungi colonized leaves tissue of halophyte (Li et al. 2020; Sun et al. 2011). Furthermore, most of the endophytic fungi isolated in this study are commonly found in various halophytic plants (Abdel Razek et al. 2020; Shreelalitha and Sridhar 2015). All isolated fungi belonged to the phylum Ascomycota, consistent with the findings of Khalil et al. (2021). In our result, the abundance of the genus was *Aspergillus*, and the abundance species was *A. flavus*. *L. axillare*, known for its resilience in harsh environments with high temperatures and limited rainfall, thrives in saline soils where fresh water for irrigation is scarce. The primary contributors to soil salinity are sodium and chloride ions, with electrical conductivity reaching up to 11% in coastal areas and up to 5% in inland regions (Bari, Yasseen, and Al‐Thani 2006; Yasseen and Al‐Thani 2013). Although the species richness of fungal endophytes from the two locations was identical, with five species identified at each site, the abundance of isolates was higher in samples collected from the coastal area. This could be attributed to the coastal region providing a more favorable habitat for the host plant. As a halophyte, *L. axillare* naturally thrives in saline soils (Alhaddad et al. 2021). Diversity indices suggest that, despite some variation in diversity and richness, the two locations share the same species composition.

The in vitro antagonistic activities of the selected fungal endophytes against different plant fungal pathogens ( *A. alternata* , *B. cinerea* , *F. oxysporum*, and *C. gloeosporioides*) showed varied effects. Antifungal efficacy was evaluated using the dual culture method against four phytopathogens, with results compared to growth on control plates. In Table 3, fungal isolate *A. terreus* (AL10) showed the highest reduction in *F. oxysporum* mycelium growth and was selected for further analysis of its secondary metabolites and potential for biocontrol under greenhouse conditions. Our findings are consistent with those of Choi and Ahsan (2022), who studied the antagonistic effect of *A. terreus* ANU‐301 against *F. oxysporum*, *Colletotrichum acutatum*, and *A. alternate*. Their results demonstrated varying levels of antifungal activity against these plant pathogens, suggesting potential broad‐spectrum control efficiency against different fungal pathogens.

The GC‐MS technique can analyze intricate mixtures and identify compounds from various classes simultaneously (Jonsson et al. 2004). Therefore, it was employed to analyze the culture filtrate of the endophytic fungi (AL 10) fungal strain. Hexadecanoic acid metabolite produced by *A. terreus* possesses an antimicrobial effect (Skanda and Vijayakumar 2021). Overall, the importance of these chemicals for plant growth would depend on their concentrations, specific effects on plant physiology, and the context of the plant's environment and growth conditions. Further research would be needed to determine their specific roles in plant growth.

*A. terreus* holds economic importance in various industries (Okabe et al. 2009). It is used in the fermentation industry for producing itaconic acid and itatartaric acid, as well as for enzyme production (Kuenz and Krull 2018). *A. terreus* exhibited cytotoxic effects against A549 human lung cancer cell lines (Goutam et al. 2017). Another study by Gupta et al. (2022) aimed to assess the biocontrol potential of the fungal endophyte *A. terreus* and its bioactive metabolite terrein, including their potential as an anticancer agent, against the ginger leaf spot phytopathogen, *C. gloeosporioides*. Ethyl acetate extracts of *A. terreus* showed insecticidal potency against larvae and pupae of some mosquitoes (Ragavendran and Natarajan 2015).

Inoculation of tomato plants with *F. oxysporum* resulted in severe wilting symptoms. However, coinoculation with the endophytic isolate *A. terreus* (AL10) significantly enhanced the resistance of tomato plants to the phytopathogen. This finding is consistent with our previous study, where tomato plant seedlings inoculated with spore suspension of *A. terreus* exhibited biocontrol and biostimulation effects on plant growth against the phytopathogen *F. oxysporum* (Alhaddad et al. 2024). Our research has shown its potential as a promising candidate for an effective biological control agent in the future.

## 5 Conclusion

Many endophytic fungi were isolated from the leaves of the halophytic plant *L. axillare*. General plants, including halophytes, are valuable sources for isolating endophytes, which show potential as growth promoters in agriculture. In‐depth systematic studies and research are needed to evaluate the effectiveness of inoculation methods using endophytes on crop plants. Future research could focus on understanding how *A. terreus* improves plant health and resilience to stress, its interactions with various plant species, and its potential applications in different agroecosystems. Evaluating the long‐term effects of *A. terreus* on soil health and ecosystems would also be valuable for its practical use in sustainable agriculture. Overall, further exploration of *A. terreus* could revolutionize agricultural strategies, leading to greater resilience and productivity.

## Author Contributions

*Conceptualization*: Fedae Alhaddad and Mohammed Abu‐Dieyeh. *Methodology*: Fedae Alhaddad and Mohammed Abu‐Dieyeh. *Software*: Fedae Alhaddad. *Validation*: Fedae Alhaddad, Mohammed Abu‐Dieyeh, Mohammad A. Al‐Ghouti, Talaat Ahmed, Samir Jaoua, and Roda Al‐Thani. *Formal analysis*: Fedae Alhaddad, Mohammed Abu‐Dieyeh, Mohammad A. Al‐Ghouti, Talaat Ahmed, Samir Jaoua, and Roda Al‐Thani. *Investigation*: Fedae Alhaddad and Mohammed Abu‐Dieyeh. *Resources*: Mohammed Abu‐Dieyeh. *Data curation*: Fedae Alhaddad, Mohammed Abu‐Dieyeh, Talaat Ahmed, Samir Jaoua, Roda Al‐Thani, and Mohammad A. Al‐Ghouti. *Writing–original draft preparation*: Fedae Alhaddad. *Writing–review and editing*: Mohammed Abu‐Dieyeh. *Visualization*: Fedae Alhaddad. *Supervision*: Mohammed Abu‐Dieyeh.

## Conflicts of Interest

The authors declare no conflicts of interest.

## Peer Review

The peer review history for this article is available in the Supporting Information for this article.

## Supporting information


**Data S1** Peer Review

## Data Availability

The data that support the findings of this study are available upon reasonable request.
