# Supplementary material for: Screening, Diversity, and Characterization of Fungal Endophytes Isolated From the Halophyte Limonium axillare and the Potential of Biocontrol Antagonists Against Fusarium oxysporum
Source: Plant Direct. 2025 Mar 24;9(3):e70026. doi: 10.1002/pld3.70026 (PMC11931262; doi:10.1002/pld3.70026)
Supplement: Supplementary file 1 — Data S1 Peer Review [file PLD3-9-e70026-s001.pdf]

Plant Direct
Open Access

Screening, diversity, and characterization of fungal endophytes isolated from the halophyte *Limonium axillare*, and the potential of biocontrol antagonists against *Fusarium oxysporum*

Fedae Alhaddad [1], Mohammed Abu-Dieyeh [1,*], Samir Jaoua [2], Mohammad A. Al-Ghouti [2], Roda Al-Thani [1], and

Talaat Ahmed [3]

Decision Letter Round 1:

September 19, 2024

Prof. Mohammed H. Abu-Dieyeh
Qatar University
Biol.& Env. Sci. Qatar University
Doha P.O.Box 27
Qatar

RE: Screening, diversity, and characterization of fungal endophytes isolated from the halophyte Limonium axillare, and the potential of biocontrol antagonists against Fusarium oxysporum

Dear Dr. Abu-Dieyeh:

Thank you for submitting to Plant Direct. All required reviews have been returned and we have now finished our evaluation of your manuscript. In light of the reviewers' comments, further revisions are needed before the paper can be accepted for publication in Plant Direct.

Please view the editors' and reviewers' comments below and use their suggestions as a guide while you work on your revision.

When uploading the revised version of this article, please be sure to include the following:

-A word document that contains your response to the reviewers. You should respond to each reviewer comment and note the changes made to the manuscript. If you do not agree with a reviewer's comment and choose not to make a suggested revision, please explain why. Please try to provide as complete an answer as possible to each reviewer's criticisms in the "response to reviewers" section.

-A tracked changes document
- A clean version of the latest version of the manuscript

To upload your revision, please click the link below.
https://plantdirect.msubmit.net/cgi-bin/main.plex?el=A3Lr2jl3A3nMc1I4A9ftddQK7UuDaQFRcjZThF3kIZwZ

In order to provide as timely a service as possible, we ask that your revision is resubmitted within three months after receipt of this request. If an extension is needed, please send a request, along with a brief explanation, to the editorial office at plantdirect@wiley.com .

Thank you very much for giving us an opportunity to review your work. I look forward to receiving the next version.

Sincerely,
Marco Giovannetti

--- Reviewer comments:
Reviewer #1:

This study examines the roles that endophytic fungi isolated from a halophyte might be able to fill in sustainable agriculture. The authors isolated fungal endophytes from the leaves of Limonium axillare, tested those fungi against Fusarium oxysporum, and then tested the ability of the endophyte to protect tomato seedlings against F. oxysporum infection.

In general, the paper could use some English language editing. It would really help with the overall flow of the paper and could help communicate some of the findings more clearly.

I think that the abstract needs to be rewritten. The organization is confusing and it is long and contains unnecessary details. Making it more concise and specific would be helpful.

The introduction would benefit from some reorganization and editing for content. There are many repetitive statements that could be edited. Here are some specific suggestions:
1. The sentence that starts on line 70 doesn't make a lot of sense to me. I don't understand the phrase "microbiomes of endophytes"

2. Line 98 is repetitive

3. The example of Piriformospora indica on line 81 may not be the most appropriate example to use in this introduction. To my knowledge, this fungus acts to promote plant growth at first, but then acts as a pathogen later.

4. The term "glycophytes" defined on line 97 seems unnecessary. Is it used later in the text?

5. The examples that start on line 107 and line 116 are redundant and in two different sections.

I have a couple of issues with the tomato seedling experiments, maybe these are a matter of clarifying how the experiments were done. However, they are important to address:
1. Were the inoculated tomato seedlings tested for colonization (of both the endophyte and pathogen)? This would be important to test because it is possible that the Aspergillus strain doesn't colonize the plant.
2. It is concerning that the control and Fusarium inoculated plants are not statistically different for most of the traits that were measured (except for chlorophyll content). I would expect that infected seedlings would be more sick.
3. I would like clarity about the timing of seedling inoculation. The way it is written right now, it seems like treatments (2) and (3) were carried out at two weeks, while the treatment (4) group was inoculated with the endophyte at two weeks and the pathogen at three weeks. If this is correct, it seems problematic that the pathogen inoculated seedlings in treatment groups (3) and (4) are different ages. This would dramatically affect the infection of the seedlings.

Reviewer #2:

The proposed article investigates the composition of the fungal endophytes isolated from a halophyte plant, L. axillare, and evaluates the capability of an isolate of A. terreus of contrasting a fungal disease in tomato plants; this work complements another publication of the same authors characterizing the bacterial community extracted from the same plant.
The authors start by sampling the plants in two locations, one in a coastal area and the other in an inland area. From the samples, they isolated different strains of fungi that were characterized at the molecular level and at the community level by considering different biodiversity indexes.
In the end, one strain was selected for its antifungal properties, analyzed in its secondary metabolite production and then tested in tomato seedling affected by the pathogenic fungus F. oxysporum, revealing a significant recovery potential when added to plants.
In general, I think that the data presented in this article are quite good but some sections could be improved in the way they are written and also some more information could be added for clarity and completeness.

Major concerns
1) The state of art displayed in the introduction delivers the important informations in a redundant and fragmented way. I believe that this whole section could be rephrased in a more logical sequence, starting from explaining the challenges faced by modern agriculture, then talking about the PGPR and the endosphere, and then concluding with halophytes and tomato. Otherwise the text results quite confusing to follow, as the single parts are not exposed linearly. For this reason, I would put the lines 87-92 at the beginning, then lines 45-63, then I would talk about the bacteria starting with lines 92-94 and then 64-86, while from line 96 on the text is clearer. Lines 72-78 can be re-written avoiding to repeat the same information. Moreover, at line 80 it is used the term "Lycopersicon esculentum" but this is outdated as it is nowadays accepted calling tomato "Solanum lycopersicum".
Line 68: "and" is not expected in this sentence and it is better to write "to increase" after "approaches".
Line 70: it is not clear what is intended with "the microbiomes of endophytes" as microbiome usually refers to a broader ecosystem.
Lines 116-117: two commas are missing after "Xylaria feejeensis", and "(halophyte)".

2) Materials and methods:
- line 134: the authors say that the plants were "thoughtfully selected" for sampling. What does this mean? How old were the plants? How far one from the other (as the distance between them could influence the biodiversity indexes)?
- wrong text formatting hides the formulas of Simpson and Shannon-Wiener indexes. I also recommend checking the spelling of the names at lines 170-177 -and also at line 356 and in

Table 2- (the correct names are Shannon-Wiener, Sorensen and Menhinick).
- greenhouse experiment section: it is not mentioned which tomato cultivar/variety was used for the experiment: is it the same as the other article (Marglobe)? This is important also to understand the life cycle of the plants and this information could be complemented by clarifying why they started the treatments on two-week old tomatoes.

3) Since the authors showed a barplot of the colonization rate, I would have also appreciated some graphical display of the biodiversity indexes.

4) I think that the results that they obtained inoculating AL10 in diseased tomatoes are really promising, but I am not so confident about the statistical tests that they carried on the data: they used ANOVA and Tukey, but with four replicates I would be more cautious and use nonparametric tests like Kruskal-Wallis and Dunn test, just to be sure not to be affected by the distribution of the data.. Or at least I would repeat the experiment again to confirm the findings.

In conclusion, I believe that this work proposes a good characterization of the fungal endophytes isolated from L. axillare. AL10 showed to be a good candidate for contrasting mycelium growth, and I really appreciated the screening of its secondary metabolites. Then, I think that the authors used a valid experimental approach to understand the effects of the endophyte in tomato, but the data can be improved in robustness by adding replicates and maybe collecting/showing physiological data of an intermediate timepoint of the experiment.
I liked the extensive discussion (even if the beginning is quite repetitive compared to the introduction), which showed how their results are supported by previous findings that allow to collocate the endophyte isolate among good candidates for in field applications.

Minor concerns:
In general, syntax and grammar can be improved, to make things clearer.
- Abstract: right at the beginning, it is not so clear if the authors are talking in general about plants living in challenging environments, or which type of plants precisely. This first sentence could be revised.
- lines 26-29 the text has a smaller dimension compared to the rest of the section
- missing space at line 40 after the full stop
- lines 262-264: the verb is missing in this sentence
- lines 328-329: I did not understand what is intended with "The taxa Aspergillus flavus (AL4) compromise the highest colonization frequency"
- line 346: better to put ":" after "species diversity"
- line 371: it is not good to write ".. it was further analyzed to analyze"

- description of Figure 5 "scan mood" instead of "scan mode"
- line 412: "chlorophyll content" is correct
- line 493: "plants" should be singular not plural
- lines 514-515: corrected sentence "Hexadecanoic Acid, another metabolite produced by A. terreus, possesses an antimicrobial effect.."

Reviewer #3:

This is an exceptionally well-written, clean manuscript nearly free of errors. The Introduction section is a bit long, but the manuscript covers a good number of diverse studies. While the findings are not especially novel, the results may translate into useful biocontrol or biofertilizer applications. The meticulous surface sterilization protocol and other methods, as well as statistical analyses were well-established and properly applied.

Two issues should be addressed before acceptance:

The authors should add some detail in the Discussion (L491-495) on the microclimate and soil characteristics at the two sites. Why were these specific sites selected? Why was a halophyte growing at the inland site? Is the soil at Location A saline? Expand upon any unique characteristics of Location B that provide an explanation for increased species diversity.

The authors should present in the Results section their findings on the list of bioactive compounds in Table 4. Some results are presented in the Discussion @ L 507-516, and so should be moved and expanded in the Results (L392). The significance or impact of the findings should be presented in the Discussion.

Some minor corrections should be addressed in a revised version of the manuscript:

L 186 Add reference for White et al. ITS primers
L 188 -190 Add sources or URL for BioEdit and MEGA L software
L198 italicize Aspergillus terreus
L211 reference or briefly describe the method "ESC 211 (Environmental Science Center) at Qatar University" for performing extraction of the bioactive 212 compounds and "applying the extracted solution to GC-MS for scan mode analysis"
L238 Add source of SPAD 238 502 (Plus) Chlorophyll meter
Fig 5 should be moved to Supplemental data

Consider combining A & B panels of Figs. 7 and 8 into single graphs with dual scales (using both sides of Y-axis) to save space. Is color needed, or could cross-hatching be used (to save cost of color reproduction)? if these figures are changed to Black and White, also consider changing retention times in Fig 5 to B&W.

References (L599-728 Please check the formatting of references- some journal titles are fully capitalized, some not. Some are missing the journal volume, some include volume and issue, some not, etc.

Decision Letter Round 2:

November 1, 2024
Prof. Mohammed H. Abu-Dieyeh
Qatar University
Biol.& Env. Sci. Qatar University
Doha P.O.Box 27
Qatar

MSID: 2024-01589R1
MS TITLE: Screening, diversity, and characterization of fungal endophytes isolated from the halophyte Limonium axillare, and the potential of biocontrol antagonists against Fusarium oxysporum

Dear Dr. Mohammed Abu-Dieyeh:

I am pleased to inform you that your manuscript "Screening, diversity, and characterization of fungal endophytes isolated from the halophyte Limonium axillare, and the potential of biocontrol antagonists against Fusarium oxysporum" has been accepted for publication in Plant Direct.

Your article will appear online in the next available issue of Plant Direct. To ensure your article gets published as quickly as possible, please pay attention to the steps detailed below. We have found that most of the delays happen at this stage, especially at the payment stage, so please respond as quickly as possible when prompted.

License Agreement: Once your article has been accepted it will move to Production and undergo admin and file checking - you may receive an email with any queries we have at this stage. When all required items are received by the publisher and queries resolved, the corresponding author will receive an email from Wiley's Author Services system which will ask them to log on at https://authorservices.wiley.com/bauthor and will present them with the appropriate license for completion. Your article cannot be published until both the signed license agreement and payment of the article fee have been received.

Payment of the Open Access Article Publication Fee: All articles published in Plant Direct are fully open access: immediately and freely available to read, download and share. Plant Direct charges a publication fee to cover the publication costs. The corresponding author for this manuscript should have already received a quote from the payments team (cs-openaccess@wiley.com) with the estimated article publication fee; please email cs-openaccess@wiley.com if this has not been received. The corresponding author should log on to the Wiley Author Services site, where the publication fee can be paid by credit card or an invoice. Pro Forma can also be requested. Payment of the publication charge must be received before the article will be published online.

Proofs: You will have the opportunity to look over your paper once more when you receive the author proofs for your article. The proofs will be with you in approximately two weeks. Please note that, in addition to publishing reviewer comments, the author's responses to review comments will also be published alongside the final version of the paper. If you would not like the author's responses to be published, please contact the editorial office at plantdirect@wiley.com .

Promotion of your article: You can help your research get the attention it deserves! Check out Wiley's free Promotion Guide for best-practice recommendations for promoting your work at www.wileyauthors.com/eeo/guide. And learn more about Wiley Editing Services which offers professional video, design, and writing services to create shareable video abstracts, infographics, conference posters, lay summaries, and research news stories for your research at www.wileyauthors.com/eeo/promotion.

Thank you again for your contribution to Plant Direct. If you have any questions, feel free to contact the editorial office at plantdirect@wiley.com .

Sincerely,

Plant Direct
Open Access

Marco Giovannetti

Esther van der Knaap

Editor, Plant Direct

-- Reviewer comments:
Reviewer #1:

Thank you for thoroughly addressing all the comments! I think it is a good manuscript.

Reviewer #3:

The authors have addressed and resolved all issues raised in the previous review to the satisfaction of this reviewer.

Response to reviewer:

Dear Editor,

Thank you for dedicating your time to assess our manuscript and offering insightful feedback. We found the remarks helpful and have included many of the suggestions in our revised manuscript. Please find our detailed responses below. Please note that the lines are related to the tracked file.

Response to Reviewer-1 Comments:

**Reviewer's comment**	**response to reviewers**
This study examines the roles that endophytic fungi isolated from a halophyte might be able to fill in sustainable agriculture. The authors isolated fungal endophytes from the leaves of *Limonium axillare*, tested those fungi against *Fusarium oxysporum*, and then tested the ability of the endophyte to protect tomato seedlings *against F. oxysporum* infection. In general, the paper could use some English language editing. It would really help with the overall flow of the paper and could help	-Thank you for your comment. The English language of the manuscript was reviewed by a person whose mother tongue is English

communicate some of the findings more clearly.	
I think that the abstract needs to be rewritten. The organization is confusing and it is long and contains unnecessary details. Making it more concise and specific would be helpful.	-Thank you for the suggestion. The abstract section has been revisited and rearranged accordingly.
The introduction would benefit from some reorganization and editing for content. There are many repetitive statements that could be edited. Here are some specific suggestions:	-Thank you for your thoughtful suggestion. We have carefully revisited the introduction and made the necessary revisions to improve the organization and reduce repetition. We believe these changes will enhance the clarity and flow of the content.
1. The sentence that starts on line 70 doesn't make a lot of sense to me. I don't understand the phrase "microbiomes of endophytes"	-Thank you for pointing that out. We have revised the sentence and removed the term 'microbiomes,' focusing solely on 'endophytes' to improve clarity. We hope this makes the statement more precise and easier to understand.
2. Line 98 is repetitive	-The sentence has been edited while keeping the focus on the key point.
3. The example of Piriformospora indica on line 81 may not be the most appropriate example to use in this introduction. To my knowledge, this fungus acts to promote plant growth at first, but then acts as a pathogen later.	-Thank you for bringing this to our attention. We have reconsidered the example of *Piriformospora indica* and have replaced it with a more suitable example in the revised introduction.
4. The term "glycophytes" defined on line 97 seems unnecessary. Is it used later in the text?	- Thank you for your suggestion. We dealt with the comment and removed the term, glycophytes.
5. The examples that start on line 107 and line 116 are redundant and in two different sections.	- Thank you for your observation. We prefer to keep both examples, as the first refers to a bacterial endophyte and the second to a fungal endophyte, despite both addressing the same pathogen (*Fusarium oxysporum*). We believe highlighting both examples is important for illustrating the differences in how bacterial and fungal endophytes can interact with the pathogen.

I have a couple of issues with the tomato seedling experiments, maybe these are a matter of clarifying how the experiments were done. However, they are important to address:	-Thank you for your comments. We did changes to address your comment.
1. Were the inoculated tomato seedlings tested for colonization (of both the endophyte and pathogen)? This would be important to test because it is possible that the Aspergillus strain doesn't colonize the plant.	-Thank you for raising this important point. In fact, we conducted a preliminary investigation by reisolating the endophyte from inoculated seedlings in the growth chamber and successfully recovered it. Additionally, during the culturing of the pathogen, we inoculated tomato seedlings to confirm that the fungus was capable of causing infection. We clarified this point by adding concise information.
2. It is concerning that the control and Fusarium inoculated plants are not statistically different for most of the traits that were measured (except for chlorophyll content). I would expect that infected seedlings would be more sick.	- Thank you for your insightful comment. We appreciate your concern regarding the statistical similarity between the control and *Fusarium*-inoculated plants for most of the measured traits, except for chlorophyll content. Yellowing of leaves in tomato is a major symptom of infection due to fusarium and not necessarily to be lethal [1]. The symptoms might be more complicated if the experiment extended in time.<br><br>[1] Keyworth, W. G. (1963). The reaction of monogenic resistant and susceptible varieties of tomato to inoculation with Fusarium oxysporum f. Zycopersici into stems or through Bonny Best rootstocks. *Annals of Applied Biology*, *52*(2), 257-270.
3. I would like clarity about the timing of seedling inoculation. The way it is written right now, it seems like treatments (2) and (3) were carried out at two weeks, while the treatment (4) group was inoculated with the endophyte at two weeks and the pathogen at three weeks. If this is correct, it seems problematic that the pathogen inoculated seedlings in treatment groups (3) and (4) are different ages. This would dramatically affect the infection of the seedlings.	-Thank you for raising this important point. The reason for inoculating the endophyte at two weeks and the pathogen at three weeks was to test the protective effect of our endophyte on the plants. We wanted to ensure that the endophyte had colonized the seedlings before they were exposed to the pathogen. If the pathogen was introduced first or at the same time, it would likely infect the seedlings, preventing the detection of the potential protective effects of the endophyte. This approach allowed us to evaluate the endophyte's ability to provide protection against *Fusarium oxysporum* and ensure the conditions necessary for proper colonization.<br><br>More clarification was added to the text. Thank you

Response to Reviewer-2 Comments:

**Reviewer's comment**	**response to reviewers**
The proposed article investigates the composition of the fungal endophytes isolated from a halophyte plant, L. axillare, and evaluates the capability of an isolate of A. terreus of contrasting a fungal disease in tomato plants; this work complements another publication of the same authors characterizing the bacterial community extracted from the same plant.<br>The authors start by sampling the plants in two locations, one in a coastal area and the other in an inland area. From the samples, they isolated different strains of fungi that were characterized at the molecular level and at the community level by considering different biodiversity indexes. In the end, one strain was selected for its antifungal properties, analyzed in its secondary metabolite production and then tested in tomato seedling affected by the pathogenic fungus F. oxysporum, revealing a significant recovery potential when added to plants.<br>In general, I think that the data presented in this article are quite good but some sections could be improved in the way they are written and also some more information could be added for clarity and completeness.	-Thank you for your kind comments. The revised version have more clarification and improvements as requested.
**Major concerns**<br>1) The state of art displayed in the introduction delivers the important informations in a redundant and fragmented way. I believe that this whole section could be rephrased in a more logical sequence, starting from explaining the challenges faced by modern agriculture, then talking about the PGPR and the endosphere, and then concluding with halophytes and tomato. Otherwise the text results quite confusing to follow, as the single parts are not exposed linearly. For this reason, I would put the lines 87-92 at the beginning, then lines 45-63, then I would talk about the bacteria starting with lines 92-94 and then 64-86, while from line	-Thank you for your valuable feedback on the structure of the introduction. We agree that a more logical flow could improve clarity. Based on your suggestions, we have updated the introduction by reorganizing the content to present the challenges in modern agriculture first, followed by a discussion of PGPR and the endosphere, and then focusing on halophytes and tomato. We also rephrased lines 72-78 to avoid repetition and ensured that the information is presented in a more linear and cohesive manner. We appreciate your insights, which have helped us enhance the overall readability of this section..

96 on the text is clearer. Lines 72-78 can be re-written avoiding to repeat the same information.<br><br>Moreover, at line 80 it is used the term "Lycopersicon esculentum" but this is outdated as it is nowadays accepted calling tomato "Solanum lycopersicum".<br><br>Line 68: "and" is not expected in this sentence and it is better to write "to increase" after "approaches".<br><br>Line 70: it is not clear what is intended with "the microbiomes of endophytes" as microbiome usually refers to a broader ecosystem.<br><br>Lines 116-117: two commas are missing after "Xylaria feejeensis", and "(halophyte)".	-Changed (page 3 line 90). Thank you<br><br>-Dealt with (page 3 line 78)<br><br>-The term microbiomes has been removed from the sentence. Page 3 line 80<br><br>-commas has been added in page 5 line 125
<u>2) Materials and methods:</u><br>- line 134: the authors say that the plants were "thoughtfully selected" for sampling. What does this mean? How old were the plants? How far one from the other (as the distance between them could influence the biodiversity indexes)?	- Thank you for your observation. By 'thoughtfully selected,' we meant that we carefully chose healthy plants to ensure consistency in our sampling. To provide more clarity, the plants were approximately [the age of the plant is difficult to be estimated as those are perennial wild plants] and were spaced one meter apart from each other. This distance was chosen to minimize any potential influence on biodiversity indexes due to proximity, ensuring that each sample reflected its unique microenvironment.

	We have updated the text to better clarify this point. page 5 line 140-143
- wrong text formatting hides the formulas of Simpson and Shannon-Wiener indexes. I also recommend checking the spelling of the names at lines 170-177 -and also at line 356 and in Table 2- (the correct names are Shannon-Wiener, Sorensen and Menhinick).	- Thanks for the correction. The correct spelling has been updated. Page 7 lines; 182,184 and 188.
- greenhouse experiment section: it is not mentioned which tomato cultivar/variety was used for the experiment: is it the same as the other article (Marglobe)? This is important also to understand the life cycle of the plants -and this information could be complemented by clarifying why they started the treatments on two-week old tomatoes.	-Yes, we used the same tomato variety in our experiments. The additional information has been added to the text. Page 8, line 226.<br><br>- Inoculating tomato seedlings at two weeks old is a common approach particularly when investigating the role of endophytes in combating pathogenic stress. At this stage, seedlings have generally developed sufficiently to benefit from the fungal association while still being in an early enough growth phase to respond positively to the fungal interaction. Inoculating seedlings at this stage allows researchers monitor interactions between endophytes and pathogens. Studies have shown that when root inoculation methods are applied to seedlings of this age, they often display faster and more pronounced pathogenic responses, making it easier to study disease progression and the mechanisms behind resistance. This early inoculation aids in gaining deeper insights into how endophytes contribute to plant defense against pathogens [1-4].<br><br>[1] Shavit, R., Ofek-Lalzar, M., Burdman, S., & Morin, S. (2013). Inoculation of tomato plants with rhizobacteria enhances the performance of the phloem-feeding insect

	Bemisia tabaci. *Frontiers in plant science*, *4*, 306.<br><br>[2] Singh, N., Phukan, T., Sharma, P. L., Kabyashree, K., Barman, A., Kumar, R., ... & Ray, S. K. (2018). An innovative root inoculation method to study Ralstonia solanacearum pathogenicity in tomato seedlings. *Phytopathology*, *108*(4), 436-442.<br><br>[3] Cason, K. T., Hussey, R. S., & Roncadori, R. W. (1983). Interaction of vesicular-arbuscular mycorrhizal fungi and phosphorus with Meloidogyne incognita on tomato. *Journal of Nematology*, *15*(3), 410.<br><br>[4] Shavit, R., Ofek-Lalzar, M., Burdman, S., & Morin, S. (2013). Inoculation of tomato plants with rhizobacteria enhances the performance of the phloem-feeding insect Bemisia tabaci. *Frontiers in plant science*, *4*, 306.
3) Since the authors showed a barplot of the colonization rate, I would have also appreciated some graphical display of the biodiversity indexes.	-Thank you for your suggestion. We appreciate your input regarding the graphical display of the biodiversity indexes. However, after careful consideration, we prefer to keep the current format to maintain consistency with the rest of the data presentation.
4) I think that the results that they obtained inoculating AL10 in diseased tomatoes are really promising, but I am not so confident about the statistical tests that they carried on the data: they used ANOVA and Tukey, but with four replicates I would be more cautious and use nonparametric tests like Kruskal-Wallis and Dunn test, just to be sure not to be affected by the distribution of the data.. Or at least I would repeat the experiment again to confirm the findings.	-Thank you for your insightful comments regarding the statistical analysis. We understand your concern about the sample size and the use of parametric tests. However, ANOVA and Tukey's tests have been demonstrated as appropriate in similar studies with comparable sample sizes [1-2] and our data met the assumptions for these tests. Furthermore, we have repeated the experiment to ensure the robustness of our findings, and the results remained consistent. We appreciate your suggestion and are

	confident in the reliability of the statistical methods used.<br><br>-A statement about the repetition of the experiment was added in the manuscript on page 8 line 232-234<br><br>[1] Palmieri, D., Vitale, S., Lima, G., Di Pietro, A., & Turrà, D. (2020). A bacterial endophyte exploits chemotropism of a fungal pathogen for plant colonization. *Nature communications*, *11*(1), 5264.<br>[2] Solaiman, M. Z., & Hirata, H. (1997). Effect of arbuscular mycorrhizal fungi inoculation of rice seedlings at the nursery stage upon performance in the paddy field and greenhouse. *Plant and Soil*, *191*, 1-12.
-In conclusion, I believe that this work proposes a good characterization of the fungal endophytes isolated from L. axillare. AL10 showed to be a good candidate for contrasting mycelium growth, and I really appreciated the screening of its secondary metabolites. Then, I think that the authors used a valid experimental approach to understand the effects of the endophyte in tomato, but the data can be improved in robustness by adding replicates and maybe collecting/showing physiological data of an intermediate timepoint of the experiment.<br><br>-I liked the extensive discussion (even if the beginning is quite repetitive compared to the introduction), which showed how their results are supported by previous findings that allow to collocate the endophyte isolate among good candidates for in field applications.	- Thank you for your kind and encouraging feedback. We are glad you found the characterization of fungal endophytes and the secondary metabolite screening of AL10 to be valuable. We appreciate your suggestion regarding increasing the number of replicates and collecting physiological data at an intermediate timepoint. These are excellent points, and we will certainly consider them in future experiments to further strengthen the robustness of the data. Your insights are greatly appreciated and will help improve our ongoing research.<br><br>- Thank you for your positive feedback on the discussion section. We are glad that you found it comprehensive and supportive of our results. Your insights are valuable and will help us improve the clarity of our work.

Minor concerns:<br>In general, syntax and grammar can be improved, to make things clearer.	- Thank you for your comments. We have revised the manuscript and further improvements have been done.
- Abstract: right at the beginning, it is not so clear if the authors are talking in general about plants living in challenging environments, or which type of plants precisely. This first sentence could be revised.	- The abstract has been revised and updated to follow a clearer structure.
- lines 26-29 the text has a smaller dimension compared to the rest of the section	-The text dimension has been changed page1 lines 32-34.
- missing space at line 40 after the full stop	-the space has been included page 2 line 45
- lines 262-264: the verb is missing in this sentence	-The verb has been added in page 10 line 284
- lines 328-329: I did not understand what is intended with "The taxa Aspergillus flavus (AL4) compromise the highest colonization frequency"	-The statement has been revised to improve clarity and flow. Page 13 lines 347-348.<br>“The species *Aspergillus flavus* (AL4) exhibited the highest colonization frequency in both locations, across all analyzed leaf segments.”
- line 346: better to put ":" after "species diversity"	-The sentence has been updated. Page 15line 367
- line 371: it is not good to write ".. it was further analyzed to analyze"	- Thanks for the correction. That sentence has been updated. Pages 15-16 lines;391-393

- description of Figure 5 "scan mood" instead of "scan mode"	-The word has been corrected.
- line 412: "chlorophyll content" is correct	-The correct word has been added. Line 435
- line 493: "plants" should be singular not plural	-The correct word has been added. Line 516
- lines 514-515: corrected sentence "Hexadecanoic Acid, another metabolite produced by A. terreus, possesses an antimicrobial effect.."	- Thanks for the correction. That sentence has been updated. Pages 23 lines;537-538

Response to Reviewer-3 Comments:

**Reviewer's comment**	**response to reviewers**
This is an exceptionally well-written, clean manuscript nearly free of errors. The Introduction section is a bit long, but the manuscript covers a good number of diverse studies. While the findings are not especially novel, the results may translate into useful biocontrol or biofertilizer applications. The meticulous surface sterilization protocol and other methods, as well as statistical analyses were well-established and properly applied.	-Thank you for your kind comments. We have tried our best to address your comment by focusing on the potential of the fungus as a biocontrol and/or biofertilizer.
Two issues should be addressed before acceptance:<br><br>(1) The authors should add some detail in the Discussion (L491-495) on the microclimate and soil characteristics at the two sites. Why were these specific sites selected? Why was a halophyte growing at the inland site? Is the soil at Location A saline? Expand upon any unique characteristics of Location B that provide an explanation for increased species diversity.	Good point to address. The analysis of the soil composition is outside the scope of this study. Previous studies have conducted an analysis on the soil and were cited accordingly. We have added more details to shed more light on the natural environment. Page22 lines (513-526)<br><br>-We have moved the paragraph to the result section as your suggestion.

(2) The authors should present in the Results section their findings on the list of bioactive compounds in Table 4. Some results are presented in the Discussion @ L 507-516, and so should be moved and expanded in the Results (L392). The significance or impact of the findings should be presented in the Discussion.	
Some minor corrections should be addressed in a revised version of the manuscript:	
L 186 Add reference for White et al. ITS primers	-The required reference has been added for the ITS primers.
L 188 -190 Add sources or URL for BioEdit and MEGA L software	-The URL has been added to the manuscript.
L198 italicize Aspergillus terreus	-The italicization has been done. Line 217
L211 reference or briefly describe the method "ESC 211 (Environmental Science Center) at Qatar University" for performing extraction of the bioactive 212 compounds and "applying the extracted solution to GC-MS for scan mode analysis"	-Arefernce has been added.
L238 Add source of SPAD 238 502 (Plus) Chlorophyll meter	-The source has been added.
Fig 5 should be moved to Supplemental data	-Thank you for your suggestion. However, we prefer to keep Fig. 5 in the main manuscript, as it provides valuable insights that allow researchers to easily explore and interpret the peaks. We believe this information is crucial for understanding the data and supporting the findings of the study.

Consider combining A & B panels of Figs. 7 and 8 into single graphs with dual scales (using both sides of Y-axis) to save space. Is color needed, or could cross-hatching be used (to save cost of color reproduction)? if these figures are changed to Black and White, also consider changing retention times in Fig 5 to B&W.	- Thank you for your suggestion. We prefer to keep the figures in color as it facilitates easier comparison between the data points. Our plan is to publish our article in a golden open access. However, if it is required by the journal to minimize reproduction costs or align with formatting guidelines, we will be happy to adjust the figures to black and white with appropriate cross-hatching. We are also open to modifying the retention times in Fig. 5 to match the journal's requirements (if needed).
References (L599-728 Please check the formatting of references- some journal titles are fully capitalized, some not. Some are missing the journal volume, some include volume and issue, some not, et	- Thank you, the references have been edited.
